# 3-*O*-Sulfated Heparan Sulfate Recognized by the Antibody HS4C3 Contribute to the Differentiation of Mouse Embryonic Stem Cells via Fas Signaling

**DOI:** 10.1371/journal.pone.0043440

**Published:** 2012-08-16

**Authors:** Kazumi Hirano, Norihiko Sasaki, Tomomi Ichimiya, Taichi Miura, Toin H. Van Kuppevelt, Shoko Nishihara

**Affiliations:** 1 Laboratory of Cell Biology, Department of Bioinformatics, Faculty of Engineering, Soka University, Hachioji, Tokyo, Japan; 2 Nijmegen Centre for Molecular Life Sciences, Department of Biochemistry, Radboud University Nijmegen Medical Center, Nijmegen, The Netherlands; Baylor College of Medicine, United States of America

## Abstract

Maintenance of self-renewal and pluripotency in mouse embryonic stem cells (mESCs) is regulated by the balance between several extrinsic signaling pathways. Recently, we demonstrated that heparan sulfate (HS) chains play important roles in the maintenance and differentiation of mESCs by regulating extrinsic signaling. Sulfated HS structures are modified by various sulfotransferases during development. However, the significance of specific HS structures during development remains unclear. Here, we show that 3-*O*-sulfated HS structures synthesized by HS 3-*O*-sulfotransferases (3OSTs) and recognized by the antibody HS4C3 increase during differentiation of mESCs. Furthermore, expression of Fas on the cell surface of the differentiated cells also increased. Overexpression of the HS4C3-binding epitope in mESCs induced apoptosis and spontaneous differentiation even in the presence of LIF and serum. These data showed that the HS4C3-binding epitope was required for differentiation of mESCs. Up-regulation of the HS4C3-binding epitope resulted in the recruitment of Fas from the cytoplasm to lipid rafts on the cell surface followed by activation of Fas signaling. Indeed, the HS4C3-binding epitope interacted with a region that included the heparin-binding domain (KLRRRVH) of Fas. Reduced self-renewal capability in cells overexpressing *3OST* resulted from the degradation of Nanog by activated caspase-3, which is downstream of Fas signaling, and was rescued by the inhibition of Fas signaling. We also found that knockdown of *3OST* and inhibition of Fas signaling reduced the potential for differentiation into the three germ layers during embryoid body formation. This is the first demonstration that activation of Fas signaling is mediated by an increase in the HS4C3-binding epitope and indicates a novel signaling pathway for differentiation in mESCs.

## Introduction

Heparan sulfate (HS) is a ubiquitous component of proteoglycans in the extracellular matrix and on the cell surface. In proteoglycans, the HS polysaccharide chains are attached covalently to Ser residues in the core proteins through the linkage region GlcAβ1-3Galβ1-3Galβ1-4Xylβ1-*O*-Ser [1]. HS chains are synthesized in the Golgi by several enzymes, including members of the EXT protein family, which elongate the HS chain by adding repeating disaccharide units of D-glucuronic acid-*N*-acetyl-D-glucosamine (-4GlcAβ1-4GlcNAcα1-)_n_. These repeating units are then modified differentially by epimerization and sulfation to produce a wide range of structurally and functionally diverse compounds [1]. Many molecules that are important for development, including members of the fibroblast growth factor (Fgf) family, Wnt, and bone morphogenic protein (BMP), can bind to specific sulfated regions of HS chains, which regulate signaling by these molecules [2], [3]. The control of sulfated HS structures is considered essential for the spatiotemporal regulation of cellular differentiation and growth throughout development.

Embryonic stem cells (ESCs) [4], [5] are promising tools for biotechnology and possess key features that should allow their exploitation in cell replacement therapies [6]. To exploit this potential, a better understanding of the molecular mechanisms that control ESC pluripotency is required. Pluripotency is regulated by a combination of intrinsic and extrinsic factors [7]. A number of these intrinsic factors have been identified, including Oct3/4 and Nanog [8]. Leukemia inhibitory factor (LIF) [9], [10], a known extrinsic factor, plays an important role in maintaining the self-renewal of mouse ESCs (mESCs) via signal transduction and activator of transcription 3 (STAT3) activation [11]. It has been reported that mESCs differentiate into primitive endoderm upon the blockage of LIF/STAT3 signaling, and Nanog inhibits this differentiation process [12], [13]. Another extrinsic factor, BMP4, acts in synergy with LIF to maintain self-renewal by modulating the Smad-mediated induction of the *Id* (*inhibitor of differentiation*) gene [14]. Wnt/β-catenin signaling also plays a role in the regulation of self-renewal that is independent of LIF/STAT3 signaling but involves Nanog expression [15]–[17]. Fgf4 is produced in an autocrine fashion in mESCs, and Fgf4/extracellular signal-regulated kinase (Erk) signaling contributes to the differentiation of mESCs into neural and mesodermal lineages [18].

Recently, we demonstrated that HS chains, including sulfated regions, contribute to the self-renewal and pluripotency of mESCs through Wnt/β-catenin and BMP/Smad signaling in culture media containing serum and LIF [16], [19]. In contrast, other groups have reported that HS chains contribute to the initiation of differentiation via Fgf4 signaling following LIF withdrawal [20], [21]. Therefore, HS chains contribute to both the maintenance of the undifferentiated state and induction of differentiation. It has also been reported that the pattern of HS chain sulfation changes during the differentiation of mESCs into mesodermal and neuroectodermal lineages [22], [23]. Specific sulfated structures in HS chains might contribute to these differentiation processes by regulating several signal transduction pathways, although the significance of the changes in the sulfation pattern of HS chains remains unclear. We suggest that alteration of HS chain sulfation patterning regulates differentiation in mESCs by controlling several signaling cascades.

3-*O*-sulfated HS structures that are recognized by the anti-HS antibody HS4C3 [24], which include GlcA/IdoA2S-GlcNS3S6S, are synthesized by HS 3-*O*-sulfotransferase (3OST), which transfers sulfate from PAPS (adenosine 3′-phosphate 5′-phosphosulphate) to the 3-OH position of a glucosamine residue to form 3-*O*-sulfated HS [25]. Six different isoforms of 3OST have been identified so far in mouse (3OST-1, -2, -3A, -3B, -5, and -6). 3-*O*-sulfated HS serves as an entry receptor for herpes simplex virus 1 (HSV-1) and binds to glycoprotein D of HSV-1 and antithrombin [26]. However, it remains unclear whether 3-*O*-sulfated HS structures, including the HS4C3-binding epitope, have other functional roles, for example within signal transduction.

In this study, we investigated the contribution of the HS4C3-binding epitope to the regulation of mESC differentiation. We found that HS4C3-binding epitope on mESCs increased after the induction of differentiation into primitive endoderm and primitive ectoderm. Up-regulation of the HS4C3-binding epitope by overexpression of *3OST* induced mESC differentiation even in the presence of LIF and serum, and demonstrated that this differentiation resulted from the redistribution of Fas to lipid rafts. In contrast, knockdown of *3OST* reduced the potential for differentiation into primitive endoderm and primitive ectoderm. The results showed that Fas signaling via the HS4C3-binding epitope contributes to general differentiation in mESCs.

## Materials and Methods

### Construction of Expression Vectors

The *3OST-2*, *-5,* and *Fas* expression vectors for transfection into mESCs were constructed using the vector pCAGIPuro (a kind gift of Prof. Kumiko Ui-Tei). The Fas ectodomain expression vectors, for the production of recombinant proteins, were constructed using the vector pGEX-6P-1 (GE Healthcare). These constructs were produced by using the GATEWAY™ cloning system (Invitrogen) as described previously [27]. Each construct contained the appropriate full-length coding sequence (*3OST-2*, amino acids 1–367; *3OST-5*, amino acids 1–346; *Fas*, amino acids 1–328) or the sequence for the putative extracellular domain of Fas, or fragments of this domain (amino acids 19–168, 19–38, and 39–168).

**Figure 1:**
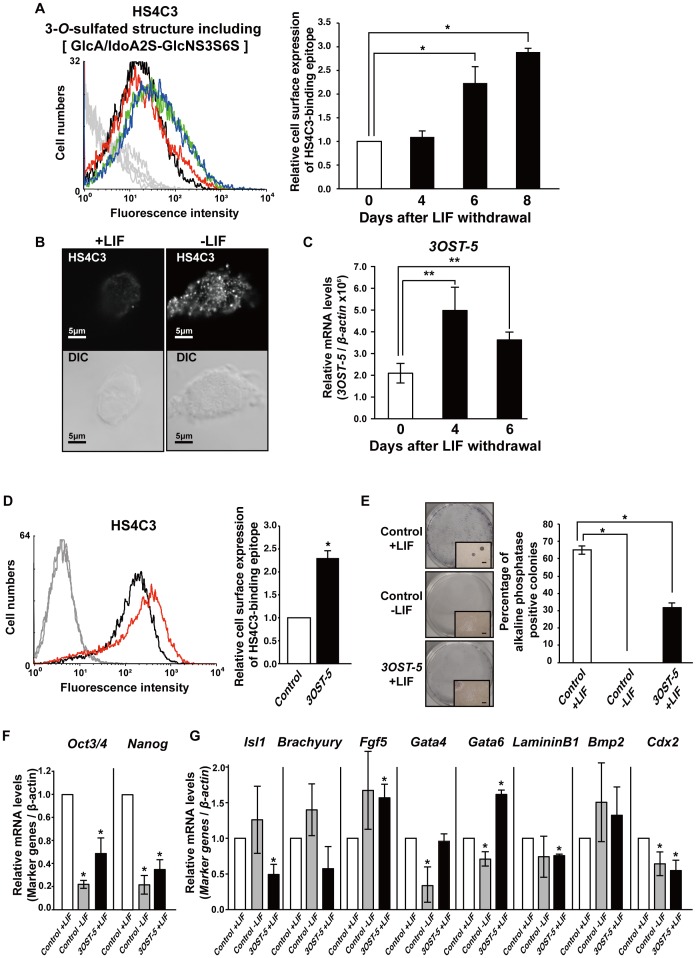
Increase in the HS4C3-binding epitope induces differentiation. (A) FACS analysis, using the HS4C3 antibody, of mESCs after LIF withdrawal up to day 8. mESCs were cultured in the presence of LIF until day 0. In the *left panel*, a histogram shows a representative result of the FACS analysis (*black line*, in the presence of LIF; *red line*, 4 days after LIF withdrawal; *green line*, 6 days after LIF withdrawal; *blue line*, 8 days after LIF withdrawal). The *gray line* shows the result obtained from cells not treated with primary antibody. In the *right panel*, the values shown are the mean fluorescence intensity ± SD after normalization against mESCs cultured in the presence of LIF (arbitrary value = 1). (B) Immunostaining, using the HS4C3 antibody, of non-permeabilized mESCs cultured in the presence of LIF (+LIF) or mESCs cultured for 7 days in the absence of LIF (−LIF) (*upper panel*). *Lower panel* shows DIC images. Scale bar, 5 µm. Representative confocal images are shown. (C) Real time PCR analysis of mESCs after LIF withdrawal up to day 6. The values shown are means ± SD. (D) FACS analysis, using the HS4C3 antibody, of mESCs at 2 days after transfection with the *3OST-5* expression construct. In the *left panel*, a histogram shows a representative result of the FACS analysis (*black line*, control cells; *red line*, cells overexpressing *3OST-5*). The *gray line* shows the result obtained from cells not treated with primary antibody. The control cells were transfected with empty pCAGI vector. In the *right panel*, the values shown are the mean fluorescence intensity ± SD after normalization against control cells (arbitrary value = 1). (E) Self-renewal assay with cells overexpressing *3OST-5*. *Left panels* show photographs of representative colonies. A high magnification image is shown at the bottom of each photograph to the right. Scale bars, 200 µm. *Right panel* shows the proportion of AP-positive colonies. The values shown are the mean ± SD. Two days after transfection, mESCs were replated in ESC medium with or without LIF. (F) and (G) Real time PCR analysis of markers of the undifferentiated (F) and differentiated state (G) in mESCs at 4 days after transfection with the *3OST-5* expression construct. The values shown are means ± SD after normalization against control cells (arbitrary value = 1). DIC, Differential interference contrast. *, *P*<0.01; **, *P*<0.05. At least three independent experiments were performed in each case.

### Cell Culture and Transfection

R1 [28] and E14TG2a [29] mESC lines were maintained on mouse embryonic fibroblasts (MEFs) inactivated with 10 µg/ml mitomycin C (Sigma) in ESC medium (DMEM supplemented with 15% FBS {Hyclone}, 1% penicillin/streptomycin {Gibco}, 0.1 mM 2-mercaptoethanol {Gibco}, and 0.1 mM non-essential amino acids {Gibco}) with 1000 U/ml LIF (Chemicon). R1 and E14TG2a lines were gifts from Dr. Seiji Hitoshi (National Institute for Physiological Sciences, Japan) and Prof. Kumiko Ui-Tei (Biophysics and Biochemistry, Graduate School of Science, University of Tokyo, Japan), respectively. MEFs were prepared from embryos at embryonic day 14.5 (E14.5; ICR). All experiments were performed in the R1 line and most were repeated using the E14TG2a line to confirm that the results were consistent.

**Figure 2:**
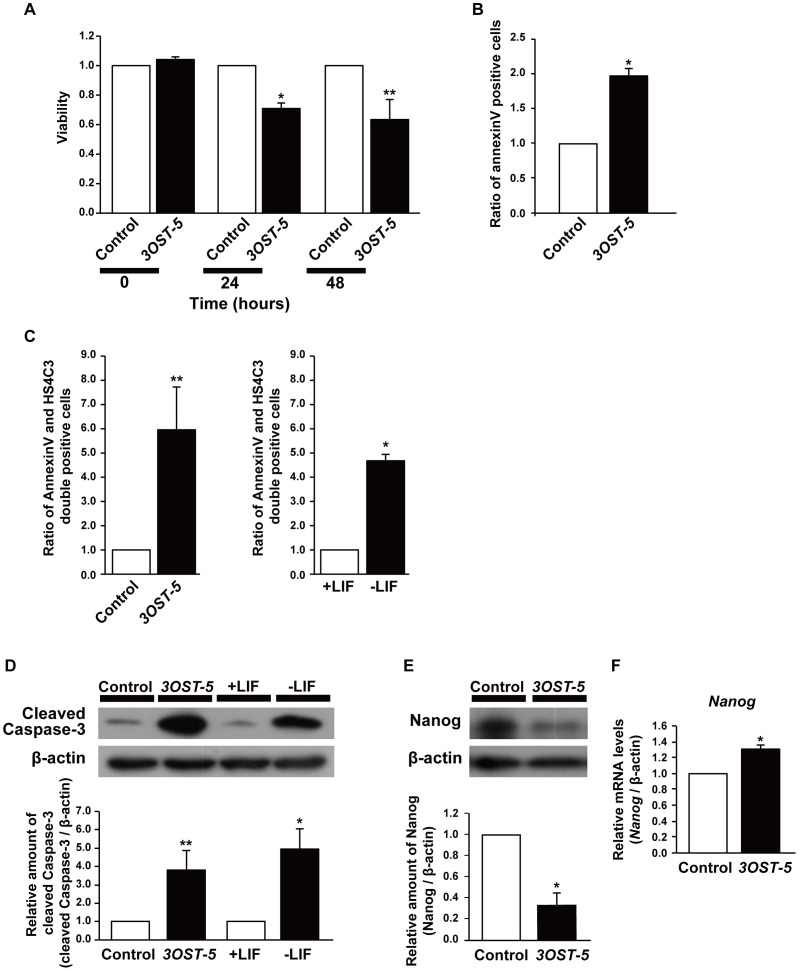
Activation of caspase-3 and degradation of Nanog are induced in cells overexpressing *3OST-5*. (A) Viability of cells overexpressing *3OST-5*. Viability was measured as described in Materials and Methods. The values shown are the means ± SD of triplicate measurements from one representative experiment after normalization against control cells (arbitrary value = 1). (B) Measurement of the rate of apoptosis in mESCs using an annexin V-FITC kit at 2 days after transfection with the *3OST-5* expression construct. The values shown are the means ± SD after normalization against control cells (arbitrary value = 1). (C) FACS analysis, using the HS4C3 antibody and annexin V, of mESCs at 2 days after transfection with the *3OST-5* expression construct (*left graph*) or of mESCs cultured for 8 days in the absence of LIF (−LIF) (*right graph*). The histograms show the ratio of the HS4C3 and annexin V double positive cells to control cells (*left graph*) or mESCs cultured in the presence of LIF (+LIF) (*right graph*) (value = 1). Values are the means ± SD. (D) and (E) Western blot analysis, using antibodies against cleaved caspase-3 or Nanog, of mESCs at 2 days after transfection with the *3OST-5* expression construct (D, *left two lanes* and E) or of mESCs cultured for 8 days in the absence of LIF (−LIF) (D, *right two lanes*). The histograms show mean densitometric readings ± SD after normalization against control cells (D, *left two lanes* and E), or mESCs cultured in the presence of LIF (+LIF) (D, *right two lanes*) (arbitrary value = 1). (F) Real time PCR analysis of *Nanog* in mESCs at 2 days after transfection with the *3OST-5* expression construct. The values shown are means ± SD after normalization against control cells (arbitrary value = 1). *, *P*<0.01; **, *P*<0.05. Three independent experiments were performed in each case.

Prior to transfection, the mESCs were harvested and 1×10^6^ cells were replated in gelatin-coated feeder-free 60-mm culture dishes (Iwaki) in ESC medium with LIF, and incubated for 16 h. Subsequently (day 1), the cells were transfected with 4 µg of pCAGI containing *3OST-2*, *3OST-5, Fas* or no insert (control) using Lipofectamine 2000 (Invitrogen). On day 2, the cells were subjected to selection with 2 µg/ml puromycin (Sigma) for 24 h. The transfection efficiency was approximately 60%, but only transfected cells survived after puromycin selection. On day 3 (2 days after transfection), the transfected cells were harvested and used in the various experiments as described below. To induce primitive endoderm, mESCs were harvested at the first and second passages and 2×10^5^ cells were replated in gelatin-coated feeder-free 60-mm culture dishes in ESC medium without LIF. At the third and fourth passages, the cells were harvested and 5×10^5^ cells were replated in gelatin-coated feeder-free 60-mm culture dishes in ESC medium without LIF. To induce embryoid body (EB) formation, the transfected cells were transferred to 60-mm Low Cell Binding dishes (Nunc) and cultured in ESC medium without LIF. To analyze the inhibition of Fas signaling, the cells were cultured in medium that contained 10 µM Ac-IETD-CHO or 20 µM Ac-DEVD-CHO (Peptide Institute Inc) dissolved in DMSO. Ac-IETD-CHO and Ac-DEVD-CHO are inhibitors of caspase-8 and caspase-3, respectively.

**Figure 3:**
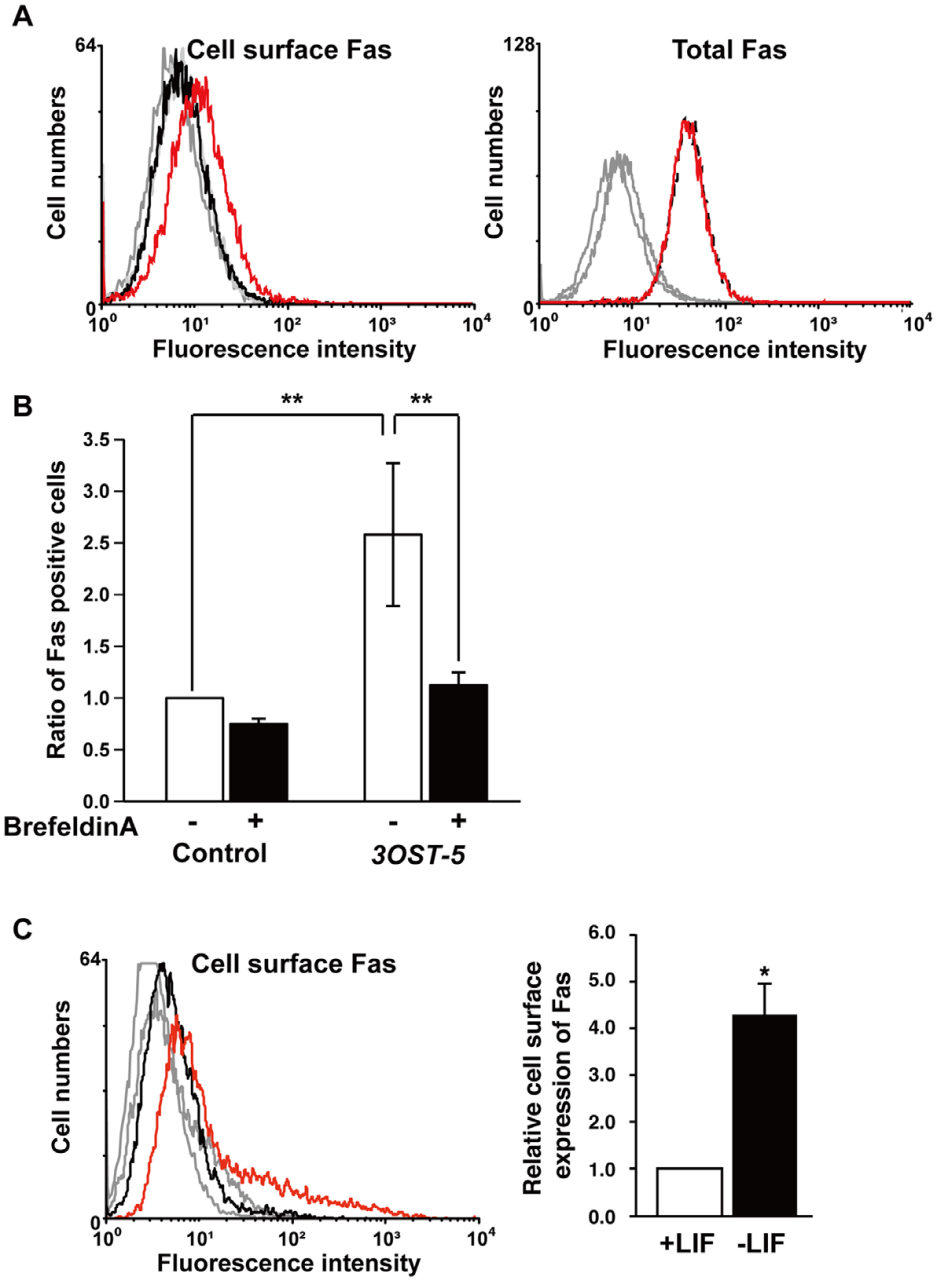
The expression of Fas increased on the surface of cells overexpressing *3OST-5*. (A) FACS analysis of cell surface Fas (*left panel)* or total Fas (*right panel*) in cells overexpressing *3OST-5* using an anti-Fas antibody (*black line*, control cells; *red line*, cells overexpressing *3OST-5*). The *gray line* shows the result obtained from cells not treated with primary antibody. As described in Materials and Methods, mESCs were permeabilized to observe total Fas expression or not permeabilized to observe Fas expression on the cell surface. (B) FACS analysis, using an anti-Fas antibody, of Fas on the surface of cells that were overexpressing *3OST-5* and had been treated with brefeldin A. The histograms show the ratio of the mean fluorescence intensity of the treated cells to that of non-treated control cells (arbitrary value = 1). Values are the means ± SD. (C) FACS analysis of cell surface Fas in mESCs cultured for 8 days in the absence of LIF using an anti-Fas antibody. In the *left panel*, a histogram shows a representative result of the FACS analysis (*black line*, in the presence of LIF; *red line*, in the absence of LIF). The *gray line* shows the result obtained from cells not treated with primary antibody. In the *right panel*, the values shown are the mean fluorescence intensity ± SD after normalization against mESCs cultured in the presence of LIF (arbitrary value = 1). *, *P*<0.01; **, *P*<0.05. Three independent experiments were performed in each case.

We generated siRNA expression plasmids that targeted *3OST-5* or *EGFP*, as a negative control, by inserting the appropriate dsDNAs between the BamHI and HindIII sites of pSilencer 3.1-H1 (Ambion) or pSUPER.retro.puro (OligoEngine). The siRNA sequences used for RNA interference (RNAi) were designed as described previously [19] using “siDirect”: EGFP, 5′-GATCCCGCCACAACGTCTATATCATGGGGAAAATCCATGATATAGACGTTGTGGCTTTTTTGGAAA-3′; *3OST-5-1*, 5′-GATCCCGTAGACCCCTCCGTCATTACCGCTTCCTGTCACGGTAATGACGGAGGGGTCTACTTTTTTA-3′; *3OST-5-2*, 5′-GATCCCCGGTTAGGACCAGCATATACAGCTTCCTGTCACTGTATATGCTGGTCCTAACCGTTTTTTA-3′.

Stable knockdown of *3OST-5* mRNA was carried out as follows. To produce retrovirus, the pSUPER.retro.puro constructs were transfected into ecotropic virus-packaging (PLAT-E) cells. Supernatants that contained virus and were derived from these PLAT-E cultures were mixed with 8 µg/ml polybrene (Sigma) and the virus/polybrene mixtures were incubated with mESCs for 24 h. After infection, the cells were replated with ESC medium containing LIF and 2 µg/ml puromycin and cultured for 5–7 days. For transient knockdown of *3OST-5* mRNA by RNAi, 4 µg of the pSilencer 3.1-H1 construct for *3OST-5* were transfected into mESCs by the method described above.

**Figure 4:**
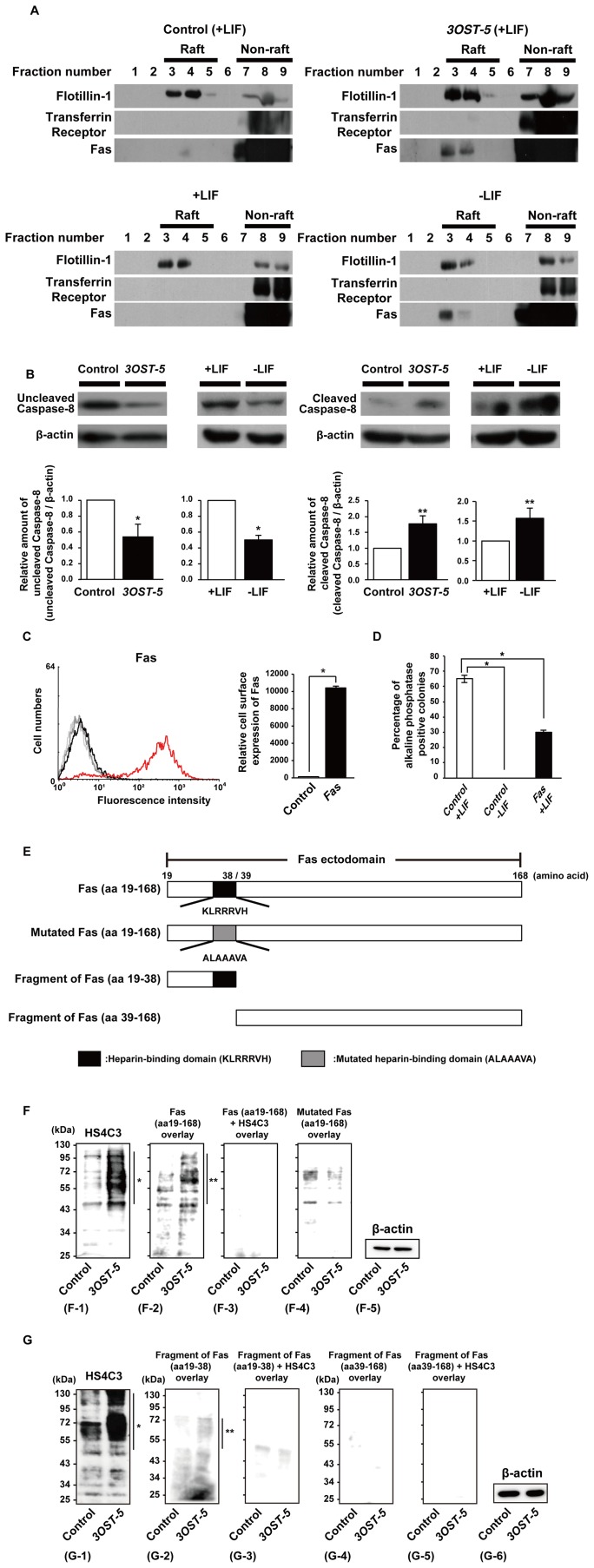
Fas signaling is activated by redistribution of Fas into lipid rafts in cells overexpressing *3OST-5*. (A) Western blot analysis of raft and non-raft fractions, using anti-Flotillin-1 (raft), anti-transferrin receptor (non-raft), and anti-Fas antibodies, of mESCs at 2 days after transfection with the *3OST-5* expression construct (*upper two panels*) or mESCs at 6 days after LIF withdrawal (*lower two panels*). At least two independent experiments were performed. Representative results are shown. (B) Western blot analysis, using antibodies against uncleaved and cleaved caspase-8, of mESCs at 2 days after transfection with the *3OST-5* expression construct (*left* and *middle right panels*) or mESCs at 8 days after LIF withdrawal (*middle left* and *right panels*). The histograms show mean densitometric readings ± SD after normalization against control cells (*left* and *middle right panels*) or mESCs cultured in the presence of LIF (*middle left* and *right panels*) (arbitrary value = 1). Three independent experiments were performed. *, *P*<0.01; **, *P*<0.05. (C) FACS analysis, using the anti-Fas antibody, of mESCs at 2 days after transfection with the *Fas* expression construct. In the *left panel*, a histogram shows a representative result of the FACS analysis (*black line*, control cells; *red line*, cells overexpressing *Fas*). The *gray line* shows the result obtained for cells that were not treated with the primary antibody. In the *right panel*, the values shown are means ± SD after normalization against control cells (arbitrary value = 1). Three independent experiments were performed. *, *P*<0.01. (D) Self-renewal assay in cells overexpressing *Fas*. The proportion of AP-positive colonies is shown. The values shown are the mean ± SD. Two days after transfection, mESCs were replated in ESC medium with or without LIF. *, *P*<0.01. (E) Mutations and truncations of the recombinant Fas ectodomain. (F) and (G) Overlay assay using the GST-fused recombinant Fas ectodomain. F-1 and G-1 show a western blot using the HS4C3 antibody. The single asterisk (*) shows the effect of the increase in the HS4C3-binding epitope on several core proteins in cells overexpressing *3OST-5*. F-2 and G-2,-4 show the overlay assay using the Fas ectodomain (F-2, aa 19–168) or fragments of the Fas ectodomain (G-2, aa 19–38; G-4, aa 39–168). F-3 and G-3,-5 show the overlay assay using the Fas ectodomain (F-3, aa 19–168) or fragments of the Fas ectodomain (G-3, aa 19–38; G-5, aa 39–168) pre-mixed with HS4C3 antibody. F-4 shows the overlay assay using the mutated Fas ectodomain (aa 19–168). The double asterisk (**) shows increased binding of the Fas ectodomain in cells overexpressing *3OST-5*. β-actin was used as a loading control for each sample (F-5 and G-6). mESCs at 2 days after transfection with the *3OST-5* expression construct were used for each analysis. Two independent experiments were performed. Representative results are shown. GST, glutathione S-transferase.

### FACS Analysis

Cells harvested 2 days after transfection were incubated with a vesicular stomatitis virus (VSV)-tagged phage-display antibody against specific sulfated HS structure [30], in FACS buffer (0.5% bovine serum albumin {BSA} and 0.1% sodium azide in PBS). After washing, the cell suspension was incubated with mouse anti-VSV glycoprotein antibody (Sigma) in FACS buffer, washed, and stained with a Cy5-conjugated anti-mouse IgG antibody (Jackson ImmunoResearch) or FITC-conjugated anti-mouse IgG antibody (Sigma) in FACS buffer. Cell analysis was performed using a FACSAria Cell Sorter (Becton Dickinson). We used the VSV-tagged HS4C3 antibody to analyze 3-*O*-sulfated HS structures [31]. We also used the anti-HS antibody 10E4 (Seikagaku Corp.) and FITC-conjugated anti-mouse IgM as the second antibody (Sigma) for antibody 10E4. We analyzed the expression of Fas using the FITC-conjugated anti-Fas antibody Jo2 (Becton Dickinson). The cells were permeabilized in 70% ethanol for 30 min on ice before Fas staining to enable analysis of total Fas expression [32]. The effect of the inhibition of protein secretion on Fas expression was examined using cells that had been treated with 5 µg/ml brefeldin A (Sigma) for 2 h before harvest.

### Measurement of Viability

Two days after transfection, cells were harvested and replated in triplicate at 0.8×10^4^ cells per well in 96-well 0.2% gelatin-coated plates in ESC medium with LIF. Cell Counting Kit-8 solution (Dojindo) was added after 0, 24 or 48 h and the cells were incubated for a further 2 h. The resulting soluble formazan product, which reflects the number of living cells, was measured at 450 nm. Viability was indicated by the ratio of the absorbance of the transfected cells to that of control cells.

**Figure 5:**
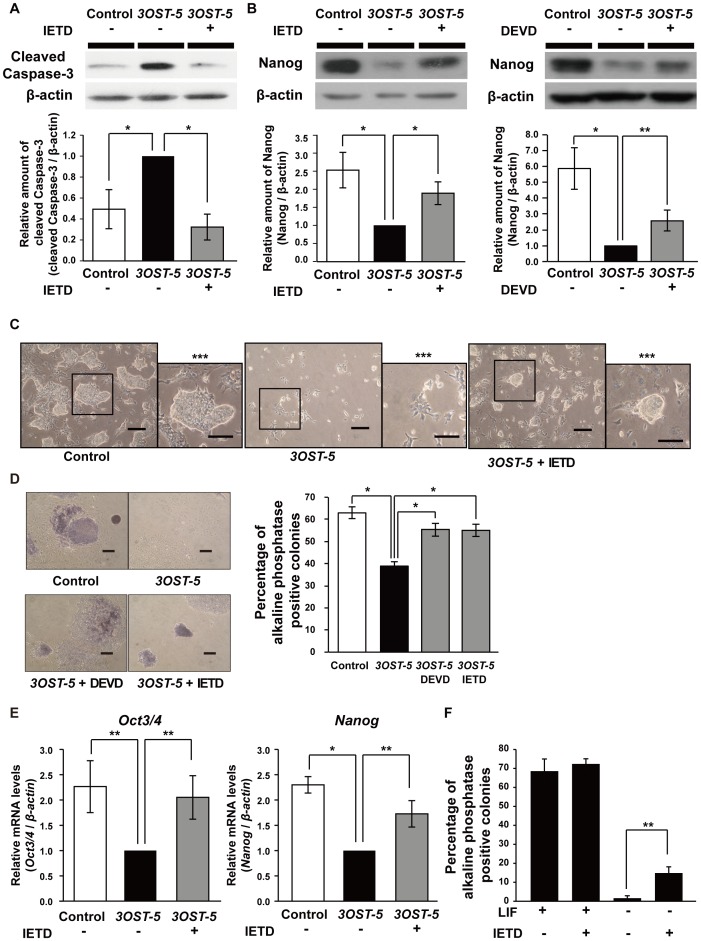
Differentiation induced by overexpression of the HS4C3-binding epitope is inhibited by an inhibitor of Fas signaling. (A) and (B) Western blot analysis using antibodies against cleaved caspase-3 and Nanog in the presence or absence of IETD or DEVD, peptides that block caspase-8 and caspase-3, respectively. Representative results are shown. The histograms show mean densitometric readings ± SD after normalization against cells overexpressing *3OST-5* but not treated with IETD or DEVD (arbitrary value = 1). mESCs were analyzed at 2 days after transfection with the *3OST-5* expression construct. (C) Representative photomicrographs of transfected cells in the presence or absence of IETD. Scale bars, 200 µm. A triple asterisk (***) indicates a high magnification image of the boxed area (Scale bars, 100 µm). mESCs were analyzed at 4 days after transfection with the *3OST-5* expression construct. (D) Self-renewal assay with cells overexpressing *3OST-5* treated with IETD or DEVD. *Left panels* show photographs of representative colonies. Scale bars, 200 µm. The *right panel* shows the proportion of AP-positive colonies. The values shown are the mean ± SD. Two days after transfection, mESCs were replated in ESC medium with LIF. mESCs were cultured with inhibitors throughout the period from transfection to AP staining. (E) Real time PCR analysis of markers of the undifferentiated state. The values shown are means ± SD after normalization against cells overexpressing *3OST-5* but not treated with IETD (arbitrary value = 1). mESCs were analyzed at 4 days after transfection with the *3OST-5* expression construct. (F) Self-renewal assay after treatment with IETD in the presence or absence of LIF. The ratio of AP-positive colonies is shown. The values shown are the mean ± S.D. IETD, Ac-IETD-CHO; DEVD, Ac-DEVD-CHO; AP, alkaline phosphatase. *, *P*<0.01; **, *P*<0.05. Three independent experiments were performed in each case.

### Measurement of Apoptotic Cells

Two days after transfection, cells were harvested and suspended in annexin V-binding buffer (BioVision). Annexin V-FITC (BioVision) was added to the cell suspensions, which were incubated at room temperature for 5 min in the dark. The suspensions were analyzed using a FACSAria Cell Sorter.

**Figure 6:**
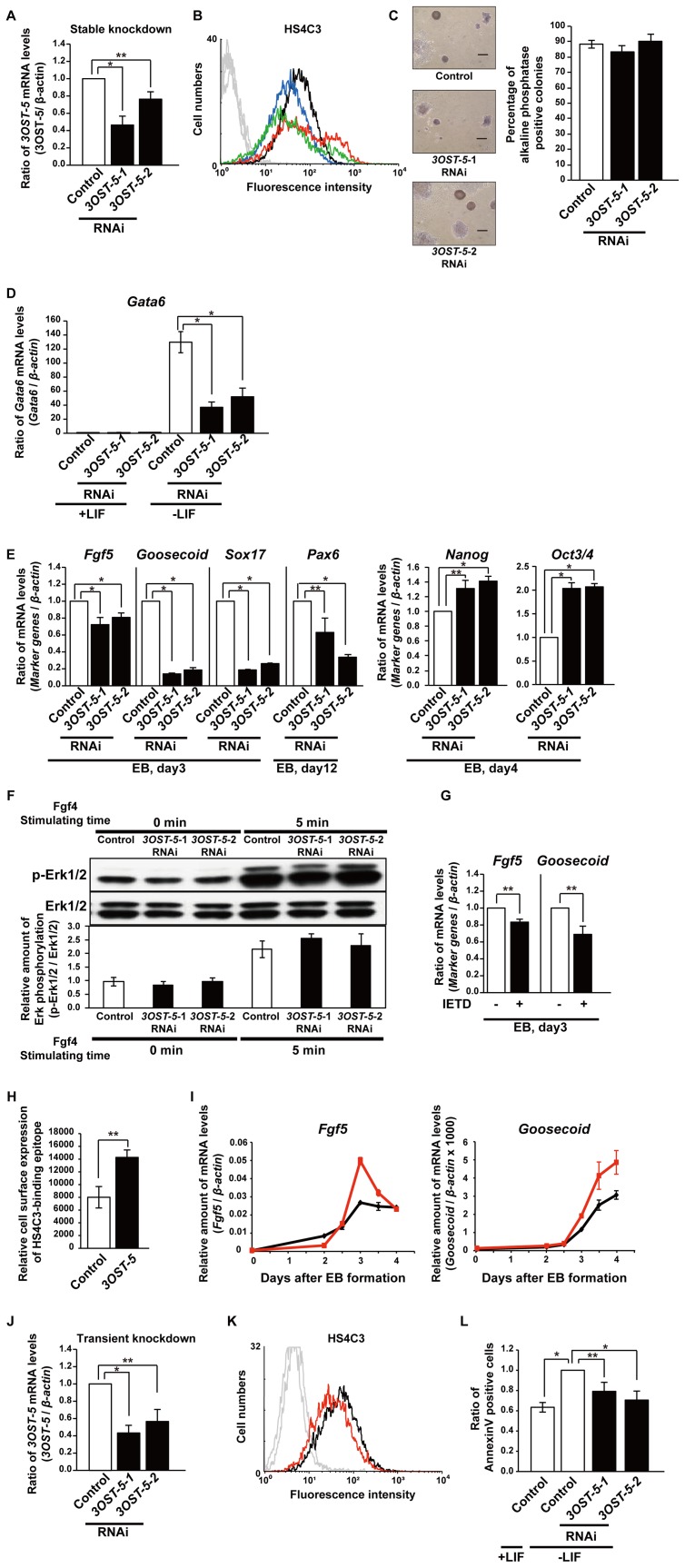
Decrease in the HS4C3-binding epitope inhibits differentiation and apoptosis. (A) and (J) Real time PCR analysis of *3OST-5* in stable (A) and transient (J) *3OST-5* KD cells. The values shown are means ± SD after normalization against control cells (arbitrary value = 1). (B) FACS analysis using the anti-HS antibody HS4C3 (*black line*, control ESCs; *blue line,* stable *3OST-5-1* KD ESCs; *red line*, control EBs; *green line*, stable *3OST-5-1* KD EBs). The control cells were transfected with the construct that targeted *EGFP*. The *gray line* shows the result obtained for cells not treated with primary antibody. (C) Self-renewal assay with stable *3OST-5* KD cells. *Left panels* show photographs of representative colonies. Scale bars, 200 µm. The *right panel* shows the proportion of AP-positive colonies. The values shown are the mean ± SD. (D) and (E) Real time PCR analysis of marker genes in stable *3OST-5* KD cells at 6 days after LIF withdrawal (D) and at 3 (*Fgf5*, *Goosecoid*, and *Sox17*), 4 (*Nanog* and *Oct3/4*), or 12 (*Pax6*) days after EB formation (E). The values shown are means ± SD after normalization against control cells (arbitrary value = 1). (F) Western blot analysis, using anti-pErk1/2 and Erk1/2 antibodies, of cells stimulated with Fgf4. The histograms show mean densitometric readings ± SD expressed as the ratio p-Erk1/2/Erk1/2. Representative results are shown. (G) Real time PCR analysis of marker genes, *Fgf5* and *Goosecoid*, in cells treated with IETD at 3 days after EB formation. The values shown are means ± SD after normalization against nontreated cells (arbitrary value = 1). (H) FACS analysis using the anti-HS antibody HS4C3 in cells overexpressing *3OST-5* at 2 days after EB formation. The values shown are mean fluorescence intensity ± SD. (I) Real time PCR analysis of *Fgf5* and *Goosecoid* in cells overexpressing *3OST-5* at 0–4 days after EB formation (*black line*, control cells; *red line*, cells overexpressing *3OST-5*). The values shown are means ± SD from duplicate measurements from one representative experiment. (K) FACS analysis using the anti-HS antibody HS4C3 (*black line*, control cells; *red line*, transient *3OST-5-2* KD cells). The *gray line* shows the result obtained for cells not treated with primary antibody. (L) Measurement of apoptosis in transient *3OST-5* KD cells using an annexin V-FITC kit at 2 days after transfection. The values shown are the means ± SD after normalization against control cells in the absence of LIF (arbitrary value = 1). KD, knockdown; RNAi, RNA interference; EB, embryoid body. *, *P*<0.01; **, *P*<0.05. Three independent experiments were performed in each case.

### Analysis of Proteins by Immunoblotting

Two days after transfection, protein samples for immunoblotting were prepared as follows. To analyze phosphorylated protein in transfected cells after extrinsic stimulation, mESC culture medium was replaced with serum-free ESC medium without LIF for 4 h and the cells were stimulated for 5, 10 or 20 min with FBS or 5 min Fgf4. Cells were then lysed with lysis buffer (50 mM Tris-HCl pH 7.4, 150 mM NaCl, 1% Triton X-100, 1 mM Na_3_VO_4_, 10 mM NaF, protease inhibitors). Lipid rafts were isolated as described previously [33], [34], [35]. Harvested cells were suspended in 0.5 ml of homogenization buffer (50 mM Tris-HCl pH 6.5, 150 mM NaCl, 5 mM EDTA, 1 mM Na_3_VO_4_, 10 mM NaF, protease inhibitors) and homogenized by passing through a 20-G needle 50 times on ice. After adding 0.5 ml of lysis buffer (50 mM Tris-HCl pH 6.5, 150 mM NaCl, 5 mM EDTA, 1.2% Triton X-100, 1 mM Na_3_VO_4_, 10 mM NaF, protease inhibitors) to the suspension, it was homogenized again by passing through a 20-G needle 10 times and incubated for 30 min on ice. The extract (approx. 1 mg of total protein) was mixed with 1.0 ml of 85% sucrose (Wako) to produce a 42.5% sucrose solution, transferred to a centrifuge tube (Beckman Coulter), and overlaid with 5 ml of 30% sucrose solution and 3 ml of 5% sucrose solution containing 50 mM Tris-HCl pH 6.5, 150 mM NaCl, and 5 mM EDTA. The discontinuous sucrose gradients were centrifuged at 4°C for 16 h in an SW41 Ti rotor (Beckman Coulter) at 30,000 rpm. The gradient was divided into nine fractions from the bottom to the top. The proteins in each fraction were precipitated with 10% trichloroacetic acid and washed with 5% trichloroacetic acid, followed by cold acetone. The precipitate was dried and dissolved in lysis buffer. Aliquots of 10 µg of total protein from the cell samples or half the total protein from each fraction were separated by 10% or 15% SDS-PAGE and transferred onto PVDF membranes (Millipore). After blocking, the membranes were incubated with antibodies against cleaved (activated) caspase-3, cleaved (activated) caspase-8, uncleaved (unactivated) caspase-8, Bad, Erk1/2, phosphorylated Erk1/2 (Thr202 and Tyr204) (Cell Signaling Technology); β-actin (Sigma); Akt, phosphorylated Akt (Ser472 and Ser473), Flotillin-1 (Becton Dickinson); Fas (M-20) (Santa Cruz); transferrin receptor (Zymed); or Nanog (ReproCELL). The membranes were then incubated with the appropriate horseradish peroxidase (HRP)-conjugated secondary antibodies (Cell Signaling Technology), washed, and developed with ECL Plus reagents (GE Healthcare).

**Figure 7:**
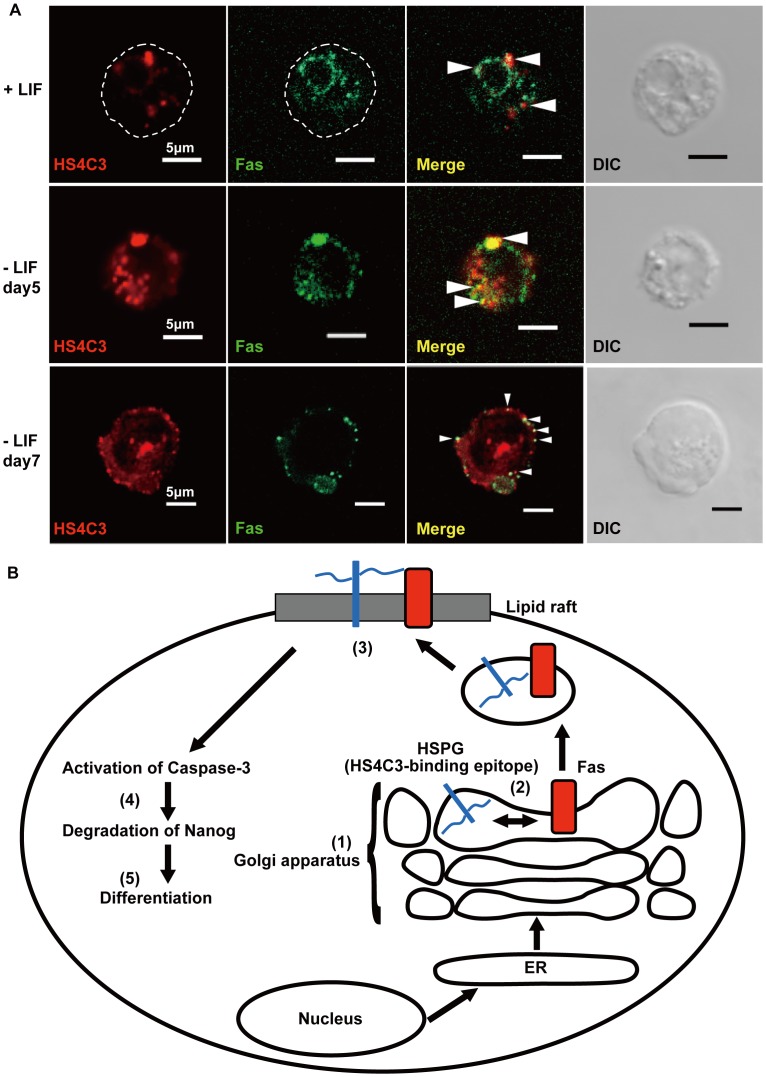
Subcellular localization of HS4C3-binding epitope and Fas, and scheme for Fas-mediated differentiation via HS4C3-binding epitope. (A) Immunostaining, using HS4C3 and anti-Fas antibodies, of permeabilized mESCs in the presence of LIF (+LIF) or mESCs cultured for 5 or 7 days in the absence of LIF (−LIF day 5 or day 7). Scale bar, 5 µm. Representative confocal images are shown. (B) Scheme for the induction of differentiation by the activation of Fas signaling via the HS4C3-binding epitope. (1) The HS4C3-binding epitope is synthesized on HSPGs in the Golgi. (2) The HS4C3-binding epitope on HSPGs interacts with Fas in the Golgi. (3) Fas then translocates into lipid rafts on the cell surface through interaction between the HS4C3-binding epitope and Fas. Fas signaling is activated by this redistribution of Fas from the Golgi into the lipid rafts. (4) Fas signaling activates caspase-3, and then activated caspase-3 degrades Nanog. (5) mESCs differentiate due to the degradation of Nanog. HSPGs, heparan sulfate proteoglycans.

### Binding Assay

The putative extracellular domain of mouse Fas or fragments thereof (amino acids 19–168, 19–38, and 39–168, lacking the signal peptide) and the domains that included point mutations (K32A, R34A, R35A, R36A, and H38A) were expressed in *Escherichia coli* BL21 cells as fusion proteins with gluthathione sepharose transferase (GST), and purified with gluthathione sepharose 4B resin (GE Healthcare) according to the manufacturer’s instructions. The K32A, R34A, R35A, R36A, and H38A point mutants were generated from the template pGEX-6P-1-Fas (amino acids 19–168) using the KOD Plus Mutagenesis Kit (Toyobo) according to the manufacturer’s instructions. Fas binding activity was examined by an overlay assay as described previously [36]. Fas overlay assays were performed on a PVDF membrane using the purified GST-Fas fusion protein. Samples (2.5 µg of total protein) were separated by 8% SDS-PAGE and then transferred to PVDF membranes. The membranes were blocked in blocking buffer (5% non-fat dry milk in PBS pH 6.5), and then incubated with 10 nM GST-Fas, GST-mutated Fas, or GST-Fas pre-mixed with HS4C3 (1∶1000) for 2 h at 4°C in PBS pH 6.5 with 3% BSA. The membranes were washed in buffer (PBS pH 6.5, 0.1% Tween 20) and incubated with HRP-conjugated anti-GST antibody (Santa Cruz) for 1 h at room temperature. Blots were developed with ECL Plus reagents.

### RT-PCR and Real Time PCR

Total RNA was isolated from cells using TRIzol® Reagent (Invitrogen) and reverse transcribed using an oligo-dT primer (Invitrogen) and a Superscript II First Strand Synthesis Kit (Invitrogen). Real time PCR was performed using an ABI PRISM® 7700 Sequence Detection System (Applied Biosystems). The relative amounts of each mRNA were normalized against the amount of *β-actin* or *GAPDH* mRNA in the same sample. Primer sets and probes for real time PCR are listed in Tables S1 and S2, respectively.

### Self-Renewal Assay

Two days after transfection of pCAGI that contained *3OST-5* or *Fas*, mESCs were replated at 1×10^4^ cells per gelatin-coated 60-mm tissue culture dish in ESC medium with or without LIF. In the case of treatment with the inhibitors IETD and DEVD, the mESCs were treated throughout the period from transfection to alkaline phosphatase (AP) staining. To detect undifferentiated cells, cells were fixed and stained with 5-bromo-4-chloro-3-indolyl phosphate-nitroblue tetrazolium (Nacalai Tesque) 5 days after replating. AP-positive colonies were counted by microscopy.

### Immunostaining of mESCs after Induction of Differentiation into Primitive Endoderm

mESCs were fixed with 4% paraformaldehyde and washed with PBS. Subsequently, the cells were blocked with buffer1 (5% BSA, 1% normal goat serum in PBS). After washing with buffer1, the cells were incubated with HS4C3 antibody in buffer1. Then, the cells were washed again and incubated with mouse anti-VSV glycoprotein antibody (Sigma). Finally, the cells were washed and costained with Cy5-conjugated anti-mouse IgG (Jackson ImmunoResearch) and the FITC-conjugated anti-Fas antibody Jo2 in buffer1. To permeabilize the cells, 0.05% Triton X-100 was added to buffer1 throughout the process. Immunofluorescence images were taken using an LSM 5 Pascal confocal laser scanning microscope (Carl Zeiss) with a 63 ×/1.3 objective at room temperature.

## Results

### Increase in HS4C3-Binding Epitope Induces Differentiation

First, we examined changes in HS chain sulfation on the surface of mESCs after induction of differentiation into primitive endoderm by LIF withdrawal. The differentiated cells displayed a reduction in the expression of *Nanog* and an increase in the expression of *Gata6*, which is a marker of the primitive endoderm (data not shown). Fluorescence-activated cell sorting (FACS) using the anti-3-*O*-sulfated HS antibody HS4C3 showed that the HS4C3-binding epitope increased steadily in differentiated mESCs (Figure 1A). Indeed, immunostaining of non-permeabilized cells showed that the HS4C3-binding epitope increased on the surface of differentiated mESCs in the absence of LIF for 7 days (Figure 1B). The expression of *3OST-5,* which has been reported to synthesize the HS4C3-binding epitope, increased when mESCs were induced to form primitive endoderm by the withdrawal of LIF (Figure 1C). Next, we performed FACS analysis of cells transfected with an expression construct for *3OST-5* using the antibody HS4C3. The HS4C3-binding epitope was increased in cells overexpressing *3OST-5*, but the amount of HS did not differ between the cells overexpressing *3OST-5* and the control cells (Figure 1D and Figure S1).

To determine whether overexpression of the HS4C3-binding epitope induced differentiation of mESCs, we performed a self-renewal assay with cells overexpressing *3OST-5*. The number of AP-positive colonies that were derived from cells overexpressing *3OST-5* decreased to approximately 30% of the number obtained with control cells even in the presence of LIF and serum in clonal density culture, which demonstrated a reduction of self-renewal in cells overexpressing *3OST-5* (Figure 1E). Then, we examined the expression of *Oct3/4* and *Nanog* in cells overexpressing *3OST-5* that were cultured with LIF; *Oct3/4* and *Nanog* are markers of the undifferentiated state. Expression of both *Oct3/4* and *Nanog* was significantly lower in cells overexpressing *3OST-5* than in control cells that were cultured with LIF (Figure 1F). Thus, overexpression of the HS4C3-binding epitope induced differentiation of mESCs. To characterize the differentiating mESCs, we evaluated the expression of several germ layer markers. In cells overexpressing *3OST-5*, we detected higher expression of markers of the primitive endoderm (*Gata6*) and primitive ectoderm (*Fgf5*) than in control cells (Figure 1G). Other lineage markers (neuroectoderm, *Isl1*; mesoderm, *Brachyury*; primitive endoderm, *Gata4*; parietal endoderm, *LamininB1*; visceral endoderm, *Bmp2*; trophoblast, *Cdx2*) were either expressed at a lower level or were unaltered as compared with control cells that were cultured with LIF (Figure 1G). These results demonstrate that the increase in the HS4C3-binding epitope induced differentiation into primitive endoderm and primitive ectoderm during culture in the presence of LIF, which should maintain the undifferentiated state.

### Activation of Caspase-3 and Degradation of Nanog are Induced in Cells Overexpressing *3OST-5*

Next, we examined cell viability because the cell number for cells overexpressing *3OST-5* was low compared with that for control cells. Indeed, cells overexpressing *3OST-5* displayed decreased viability, which might be caused by apoptosis (Figure 2A). Cell differentiation and apoptosis are linked, and mESCs undergo caspase-mediated apoptosis during differentiation induced by LIF withdrawal [37]. As shown in Figure 2B, the proportion of annexin V-positive cells increased significantly in cells overexpressing *3OST-5* as compared with control cells. In addition, the ratio of annexin V and HS4C3 double-positive cells was approximately five times higher in cells overexpressing *3OST-5* and mESCs induced to differentiate by culture in the absence of LIF for 8 days than in control cells (Figure 2C). These results indicate that overexpression of the HS4C3-binding epitope induced both differentiation and apoptosis of mESCs in a similar manner to LIF withdrawal.

Caspase-3 is activated during mESC differentiation (Figure 2D, [37]). Therefore, we investigated whether caspase-3 is activated in cells overexpressing *3OST-5* and found that its level of activation was indeed higher in these cells than in control cells (Figure 2D). Given that activated caspase-3 degrades Nanog, which results in mESC differentiation [38], we compared the amounts of Nanog in cells overexpressing *3OST-5* and control cells. The amount of Nanog protein was decreased in cells overexpressing *3OST-5* (Figure 2E), whereas the level of *Nanog* mRNA was not decreased (Figure 2F). These results suggest that induction of differentiation in cells overexpressing *3OST-5* depends on the activation of caspase-3 followed by the degradation of Nanog.

### The Expression of Fas Increased on the Surface of Cells Overexpressing *3OST-5*

To clarify the mechanism by which caspase is activated during mESC differentiation, we investigated the contribution of upstream components of the caspase cascade. Fas (CD95 or Apo-1) is a type I membrane protein and a member of the tumor necrosis factor receptor family. Fas signaling, which is activated by interaction between Fas and the Fas ligand (FasL) or simply by the redistribution of Fas into lipid rafts, comprises part of the caspase activation cascade [39], [40]. First, we determined the distribution of Fas on cells overexpressing *3OST-5*. Fas was present at a very low level on the surface of control cells but this level was clearly higher on cells overexpressing *3OST-5* (Figure 3A left histogram and 3B). However, the total level of Fas did not change between the two cell types (Figure 3A right histogram). The increase in the level of Fas on the surface of cells overexpressing *3OST-5* was inhibited by treating the cells with brefeldin A, which inhibits the transport of proteins from the ER to the Golgi and leads to the accumulation of proteins inside the ER (Figure 3B). Furthermore, the level of Fas on the surface of mESCs differentiated by culture in the absence of LIF for 8 days was increased (Figure 3C).

### Overexpression of the HS4C3-Binding Epitope Recruits Fas into Lipid Rafts, which Activates Fas Signaling

Next, we analyzed biochemically whether Fas was localized in lipid rafts in differentiated cells. The separation of lipid rafts and non-rafts was confirmed by western blotting using Flotillin-1 as a marker of the raft fraction (fractions 3–5) and the transferrin receptor as a marker of the non-raft fraction (fractions 7–9) (Figure 4A). Fas was located in the raft fraction in cells overexpressing *3OST-5* in the presence of LIF (+LIF) and in mESCs differentiated by culture in the absence of LIF for 6 days (−LIF) (Figure 4A), which indicated that the activation of Fas signaling in these cells was mediated by the redistribution of Fas into lipid rafts. In addition, the HS4C3-binding epitope was localized in lipid rafts in cells overexpressing *3OST-5* (Figure S2). Caspase-8 is a downstream component of the Fas signaling pathway. We also found that the level of activated caspase-8 was increased, and the level of unactivated caspase-8 decreased, in cells overexpressing *3OST-5* and in mESCs after the induction of differentiation by LIF withdrawal for 8 days (Figure 4B). These data indicated that Fas signaling had been activated.

To determine whether Fas signaling induced the differentiation of mESCs, we performed a self-renewal assay with cells overexpressing *Fas*. FACS analysis showed that the amount of Fas on the cell surface was increased in cells overexpressing *Fas* (Figure 4C). The number of AP-positive colonies that were derived from cells overexpressing *Fas* was reduced significantly to the same number as those derived from cells overexpressing *3OST-5*, even in the presence of LIF and serum in clonal density culture (Figure 4D). This indicated that up-regulation of Fas reduced the self-renewal of mESCs.

We examined the putative interaction of Fas and the HS4C3-binding epitope by using an overlay assay. Given that we expected the heparin-binding domain (KLRRRVH) in the Fas ectodomain to bind in the Golgi to sulfated regions of HS chains, including the HS4C3-binding epitope, we used several types of the recombinant Fas ectodomain for the assay (Figure 4E). As shown in Figure 4F (F-2), overlaid Fas (indicated by ** in the figure) increased in parallel with the HS4C3-binding epitope (indicated by * in Figure 4F (F-1)) in cells overexpressing *3OST-5*, which indicated that the Fas ectodomain bound specifically to the HS4C3-binding epitope. Addition of HS4C3 blocked Fas binding completely, which confirmed the interaction was specific (Figure 4F (F-3)). Furthermore, point mutations in the heparin-binding domain (ALAAAVA) of Fas abolished the binding (Figure 4F (F-4)). In addition, we prepared two separate fragments of the Fas ectodomain, amino acids 19–38 and 39–168, (Figure 4E) and performed the overlay assay with these fragments. Binding of the fragment that comprised amino acids 19-38, which included the heparin-binding domain, was increased in cells overexpressing *3OST-5* (Figure 4G (G-2)). In addition, the fragment that comprised amino acids 39-168 did not bind to samples from control cells and cells overexpressing *3OST-5* (Figure 4G (G-4)). Addition of HS4C3 also blocked fragments binding (Figure 4G (G-3, G-5)). Therefore, we concluded that amino acids 19–38 of Fas, which include the heparin-binding domain, are necessary for interaction with the HS4C3-binding epitope. These findings demonstrated for the first time that Fas binds to HS chains, including the HS4C3-binding epitope, through the region that contains the heparin-binding domain (KLRRRVH).

Another important caspase cascade is mediated by changes in mitochondrial permeability, which are regulated by proapoptotic Bcl-2 proteins such as Bad. Bad is phosphorylated as a result of the activation of signal transduction by several survival factors, including growth factors. We examined the phosphorylation of Akt, which is upstream of Bad, after serum stimulation and observed a similar increase in the level of phosphorylated Akt in control cells and cells overexpressing *3OST-5* (Figure S3A). Interestingly, the expression of Bad was markedly decreased in cells overexpressing *3OST-5*, which indicated a reduction in the Bad-mediated proapoptotic state (Figure S3B). These results demonstrate that activation of caspase-3 in cells overexpressing *3OST-5* was not due to a defect in the mitochondrial pathway.

Taken together, our findings provide the first demonstration that redistribution of Fas from intracellular pools to lipid rafts on the cell surface depends on an interaction between Fas and the HS4C3-binding epitope, and is followed by the activation of Fas signaling and caspase-3.

### Differentiation of mESCs Induced by Overexpression of the HS4C3-Binding Epitope is Inhibited by Blockage of Fas Signaling

To confirm that activation of Fas signaling via overexpression of the HS4C3-binding epitope was involved in mESC differentiation, we investigated whether the reduced self-renewal capability of cells overexpressing *3OST-5* could be rescued by blocking Fas signaling using the peptides Ac-IETD-CHO (IETD) and Ac-DEVD-CHO (DEVD), which block the activity of caspase-8 and caspase-3, respectively. We found that treatment of cells overexpressing *3OST-5* with the inhibitors inhibited caspase-3 activation and rescued the degradation of Nanog protein (Figure 5A and 5B). Next, we compared the morphologies of control cells and cells overexpressing *3OST-5*. Control cells had an undifferentiated appearance with a moderately packed morphology (Figure 5C). In contrast, almost all cells overexpressing *3OST-5* had a flattened, differentiated morphology (Figure 5C). Some IETD-treated cells had a similar morphology to undifferentiated mESCs (Figure 5C). Then, we performed a self-renewal assay and counted the AP-positive colonies. Treatment of cells overexpressing *3OST-5* with DEVD or IETD restored the proportion of AP-positive colonies to a level similar to that obtained with control cells (Figure 5D). Furthermore, the level of *Oct3/4* and *Nanog* mRNA in cells overexpressing *3OST-5* was higher after IETD treatment than in untreated cells (Figure 5E). Thus, we demonstrated that activation of Fas signaling via overexpression of the HS4C3-binding epitope induced differentiation in mESCs. As shown in Figure 4B, Fas signaling was activated in mESCs after the induction of differentiation by LIF withdrawal. We expected that blocking Fas signaling would inhibit the induction of differentiation by LIF withdrawal. Indeed, resistance to differentiation caused by activation of Fas signaling was observed in mESCs treated with IETD in the absence of LIF (Figure 5F). These results indicated that Fas signaling induces the differentiation caused by LIF withdrawal in mESCs. Taken together, rescue experiments that involved blocking Fas signaling demonstrated that the degradation of Nanog protein and induction of differentiation were actually caused by Fas signaling via HS4C3-binding epitope.

### Decrease in the HS4C3-Binding Epitope Inhibits Differentiation and Apoptosis

To examine and confirm the requirement for HS4C3-binding epitope for the differentiation of mESCs, we performed stable and transient knockdown (KD) of *3OST-5* mRNA using RNAi. We designed two constructs that targeted *3OST-5* (*3OST-5-1* and *3OST-5-2*, which expressed different siRNAs targeting *3OST-5*) and one that targeted *EGFP* as a negative control. The level of *3OST-5* expression was reduced in both stable and transient *3OST-5* KD cells (Figure 6A and 6J). FACS analysis showed that the HS4C3-binding epitope was decreased in both stable and transient *3OST-5* KD cells (Figure 6B and K). Then, we performed a self-renewal assay with the stable *3OST-5* KD cells. The number of AP-positive colonies did not differ between the stable *3OST-5* KD cells and the control cells in the presence of LIF and serum in clonal density culture (Figure 6C). Furthermore, the expression of markers of the undifferentiated and differentiated states did not change even in the stable *3OST-5* KD cells (Figure S4). These results demonstrated that the reduction in the HS4C3-binding epitope did not affect the self-renewal capability of mESCs.

To determine whether down-regulation of the HS4C3-binding epitope affected the potential of mESCs for differentiation, stable *3OST-5* KD cells were induced to form primitive endoderm by LIF withdrawal for 6 days. In the stable *3OST-5* KD cells, the increase in the expression of *Gata6* (primitive endoderm marker) that was seen in the control cells was inhibited (Figure 6D). This finding indicated that the HS4C3-binding epitope was necessary for differentiation into primitive endoderm. Next, we investigated *in vitro* differentiation into embryoid bodies (EBs), which comprise three germ layers: endoderm, mesoderm, and ectoderm. Expression of the HS4C3-binding epitope was increased during EB formation in control cells (Figure 6B). In EBs derived from stable *3OST-5* KD cells, HS4C3-binding epitope was decreased compared with that in control EBs (Figure 6B). In turn, the expression of *Fgf5* (primitive ectoderm marker), *Goosecoid* (mesoderm marker), *Sox17* (endoderm marker), and *Pax6* (ectoderm marker) was decreased by down-regulation of the HS4C3-binding epitope (Figure 6E). Furthermore, *Nanog* and *Oct3/4* were expressed at a higher level in stable *3OST-5* KD cells than in control cells at 4 days after EB formation (Figure 6E). These data demonstrated that differentiation into all three germ layers was inhibited by down-regulation of the HS4C3-binding epitope during EB formation. Then, we examined Fgf4/Erk signaling, which is reported to be a trigger of stem cell differentiation [18], and observed no differences in the level of phosphorylated Erk1/2 between stable *3OST-5* KD cells and control cells after exposure to Fgf4 (Figure 6F). Hence, Fgf4/Erk signaling did not contribute to the reduction of the potential for differentiation in stable *3OST-5* KD cells. Given the result obtained in the present study that the HS4C3-binding epitope contributed to Fas signaling during the differentiation of mESCs into primitive endoderm, we expected that Fas signaling would also function during EB formation. Therefore, we used IETD, a caspase-8 inhibitor, to analyze the role of Fas signaling in EB differentiation. Treatment with IETD throughout EB differentiation led to a reduction in the expression of *Fgf5* (primitive ectoderm marker) and *Goosecoid* (mesoderm marker), which indicated that Fas signaling was necessary for EB differentiation (Figure 6G). The findings demonstrated that Fas signaling via the HS4C3-binding epitope induced normal EB differentiation. In addition, in EBs derived from cells overexpressing *3OST-5*, expression of the HS4C3-binding epitope, *Fgf5,* and *Goosecoid* were increased as compared with EBs derived from control cells (Figure 6H and 6I). Furthermore, as shown in Figure 6L, the population of annexin V-positive cells was increased in control cells after LIF withdrawal for 24 hours, whereas that of annexin V-positive cells did not increase in response to LIF withdrawal in *3OST-5* transient KD cells. These data showed that Fas signaling via the HS4C3-binding epitope was indispensable for the induction of apoptosis and differentiation of mESCs into primitive endoderm and EBs.

### Fas Colocalizes with HS4C3-Binding Epitope in mESCs

We examined the localization of HS4C3-binding epitope and Fas in mESCs during differentiation in response to LIF withdrawal. In the undifferentiated state in the presence of LIF, confocal slices showed that the HS4C3-binding epitope was colocalized with Fas in the intracellular Golgi compartment around the nucleus, not on the cell surface (Figure 7A, indicated by the arrowheads in the upper panels). However, in mESCs induced to differentiate by culture in the absence of LIF for 5 or 7 days, expression of the HS4C3-binding epitope increased, and colocalization of the HS4C3-binding epitope and Fas was observed as dots on the cell surface (Figure 7A, indicated by the arrowheads in the middle and lower panels). These images were consistent with the increase in HS4C3-binding epitope and Fas on the surface of differentiated cells that was shown by the FACS analysis in Figure 3A, and supported the redistribution of Fas into lipid rafts that was indicated by the biochemical analysis in Figure 4A. Taken together, the results demonstrated that Fas, which was localized to the Golgi in the undifferentiated state, was shifted to lipid rafts on the cell surface by binding to the HS4C3-binding epitope during differentiation.

## Discussion

Herein we report for the first time that activation of Fas signaling via the HS4C3-binding epitope induced the differentiation into primitive endoderm and primitive ectoderm from mESCs. From our results, we propose the following scheme (Figure 7B). The level of the HS4C3-binding epitope in mESCs increased during differentiation (Figure 1A, 1B, and Figure 6B). The HS4C3-binding epitope interacted with the heparin-binding domain (KLRRRVH) of Fas in the Golgi, which resulted in the redistribution of Fas to lipid rafts and the activation of Fas signaling. Activation of Fas caused degradation of Nanog via activated caspase-3, which induced differentiation (Figure 2D and 2E). The proposed scheme was supported by rescue experiments in which Fas signaling was blocked (Figure 5) and by confocal slices (Figure 7A). Therefore, we propose that the activation of Fas signaling mediated by the HS4C3-binding epitope is a novel mechanism for the regulation of differentiation in mESCs.

It has been reported that Fas is transported from intracellular pools to the cell surface in response to various stimuli [32],[41]. However, the identities of the molecules that contribute to this transport of Fas have yet to be determined. Two possible transport pathways have been implicated. 1) The intracellular domain of secreted proteins might interact with Fas in the cytoskeletal network and recruit Fas from the cytoskeletal network to the cell surface. Ivanov *et al.* reported that Fas-associated phosphatase-1 (FAP-1) contributes to the localization of Fas by binding via the intracellular domain of Fas within the cytoskeletal network [41]. 2) The extracellular domain of secreted proteins might interact with Fas in the Golgi lumen and recruit Fas from the Golgi to the cell surface. Our proposed scheme corresponds to this second possible pathway. As shown in Figure 3B, the effect of brefeldin A on the translocation of Fas was probably due to the inhibition of secretion of HS proteoglycans (HSPGs) that contained the HS4C3-binding epitope. Recently, it has been demonstrated that Fas is redistributed from intracellular pools to lipid rafts on the cell surface in Swiss 3T3 cells overexpressing *syndecan-2* [42]. However, the mechanism of this redistribution has not yet been clarified. We obtained data that demonstrated that syndecan-2 was localized to lipid rafts in mESCs (data not shown). Considering our results and the above reported findings, we propose that HS chains on syndecan-2 interact with Fas in the Golgi lumen and mediate the transport of Fas from the Golgi to the cell surface, that is, via pathway 2) described above.

Gabriella *et al.* have reported that FasL is not expressed in undifferentiated mESCs [43]. Our preliminary studies showed that expression of *FasL* was not increased in mESCs during differentiation induced by LIF withdrawal or in cells overexpressing *3OST-5* (Figure S5). In tumor cells, Fas signaling is amplified by the redistribution of Fas into lipid rafts alone, even if FasL does not bind to Fas [39]. Therefore, the data suggest that, in cells overexpressing *3OST-5*, Fas signaling can be activated independently of Fas/FasL interaction simply by redistributing Fas into lipid rafts.

The *in vitro* activities of mouse 3OSTs have not been reported yet. However, it has been reported that the human 3OST-2, 3, 4, and 5 isoforms synthesize the HS4C3-binding epitope [25]. Furthermore, among human 3OST-2, 3, 4, and 5, 3OST-2 and -5 correspond clearly to mouse 3OST-2 and 3OST-5, respectively. We performed FACS analysis using the HS4C3 antibody and confirmed that the HS4C3-binding epitope increased in cells overexpressing *3OST-2* or *-5* (Figure S6A). In *3OST-2*-transfected cells, we obtained the same results as in *3OST-5*-transfected cells (Figure S6A–C). These data indicated that 3OST-2 also activated Fas signaling and induced the differentiation.

As shown in Figure 1G, cells overexpressing *3OST-5* had the potential to differentiate not only into primitive endoderm but also into primitive ectoderm. On the other hand, stable *3OST-5* KD cells lost the potential to differentiate into primitive endoderm and primitive ectoderm (Figure 6A–E). Furthermore, treatment with IETD, an inhibitor of caspase-8, interfered with normal differentiation into primitive endoderm and primitive ectoderm (Figure 5F and 6G). Therefore, the activation of Fas signaling via the HS4C3-binding epitope, which was indicated in this study, is involved in the differentiation of mESCs into both primitive endoderm by LIF withdrawal and primitive ectoderm by EB formation. We showed clearly that Fas signaling functioned during these differentiation processes. Interestingly, although Fgf4/Erk signaling was functioning, differentiation into primitive endoderm, primitive ectoderm, and subsequently all three germ layers was inhibited in the stable *3OST-5* KD cells. Therefore, a signal other than Fgf4/Erk signaling must function to induce differentiation. Hence, in addition to Fgf4/Erk signaling, we propose that the activation of Fas signaling via the HS4C3-binding epitope plays an important role in the early stages of all types of differentiation in mESCs.

## Supporting Information

Figure S1 **The expression of HS in cells overexpressing** ***3OST-5*** **.** FACS analysis using the anti-HS antibody 10E4 (*black line*, control cells; *red line*, cells overexpressing *3OST-5*). *The gray line* shows the result obtained for cells not treated with primary antibody.
(TIF)

Figure S2 **HS4C3-binding epitope localized in lipid rafts in cells overexpressing** ***3OST-5*** **.** Western blot analysis of raft and non-raft fractions using anti-Flotillin-1 (raft), anti-transferrin receptor (non-raft), and HS4C3 antibodies. Representative results are shown.
(TIF)

Figure S3 **Mitochondrial pathways mediated by survival factors are not affected in cells overexpressing** ***3OST-5*** **.** (A) and (B) Western blot analysis, using antibodies against p-Akt, Akt, and Bad, of cells stimulated with FBS. The histograms show mean densitometric readings ± SD for the ratio p-Akt/Akt or Bad/β-actin. Three independent experiments were performed, and representative results are shown.
(TIF)

Figure S4 **Pluripotency was maintained in stable** ***3OST-5*** **knockdown cells.** Real time PCR analysis of markers of the undifferentiated and differentiated states in stable *3OST-5* knockdown cells. The values shown are means ± SD after normalization against control cells (arbitrary value = 1). Three independent experiments were performed. *, *P*<0.01.
(TIF)

Figure S5 **The expression of** ***Fas ligand*** **was not increased in cells overexpressing** ***3OST-5*** **.** RT-PCR analysis of the expression of *Fas ligand* in cells overexpressing *3OST-5*. *GAPDH*, glyceraldehyde-3-phosphate dehydrogenase.
(TIF)

Figure S6 **The overexpression of** ***3OST-2*** **also activated Fas signaling and induced the differentiation of mESCs.** (A) FACS analysis, using the HS4C3 antibody, of mESCs at 2 days after transfection with the *3OST-2* expression construct (*black line*, control cells; *green line*, cells overexpressing *3OST-2*). The *gray line* shows the result obtained from cells not treated with primary antibody. (B) Real time PCR analysis of markers of the undifferentiated state in cells overexpressing *3OST-2*. The values were normalized against control cells (arbitrary value = 1). (C) western blot analysis using an antibody against uncleaved caspase-8, cleaved caspase-3, or Nanog. The histograms show mean densitometric readings ± SD after normalization against control cells (arbitrary value = 1). **, P<0.05. Three independent experiments were performed.
(TIF)

Table S1 **List of gene specific primers for real time PCR.**
(TIF)

Table S2 **List of gene specific probes for real time PCR.**
(TIF)
