# Correction: 3-*O*-Sulfated Heparan Sulfate Recognized by the Antibody HS4C3 Contribute to the Differentiation of Mouse Embryonic Stem Cells via Fas Signaling

**DOI:** 10.1371/annotation/dc398db7-e121-4bce-82d1-feac6d9bc68f

**Published:** 2012-10-24

**Authors:** Kazumi Hirano, Norihiko Sasaki, Tomomi Ichimiya, Taichi Miura, Toin H. Van Kuppevelt, Shoko Nishihara

The word "Contributes" is misspelled in the article title. The correct title is: 3-*O*-Sulfated Heparan Sulfate Recognized by the Antibody HS4C3 Contributes to the Differentiation of Mouse Embryonic Stem Cells via Fas Signaling.

The correct citation is: Hirano K, Sasaki N, Ichimiya T, Miura T, Van Kuppevelt TH, et al. (2012) 3-*O*-Sulfated Heparan Sulfate Recognized by the Antibody HS4C3 Contribute to the Differentiation of Mouse Embryonic Stem Cells via Fas Signaling. PLoS ONE 7(8): e43440. doi:10.1371/journal.pone.0043440 

