# Supplementary figures and images for: 3-O-Sulfated Heparan Sulfate Recognized by the Antibody HS4C3 Contribute to the Differentiation of Mouse Embryonic Stem Cells via Fas Signaling
Source: PLoS One. 2012 Aug 16;7(8):e43440. doi: 10.1371/journal.pone.0043440 (PMC3420900; doi:10.1371/journal.pone.0043440)

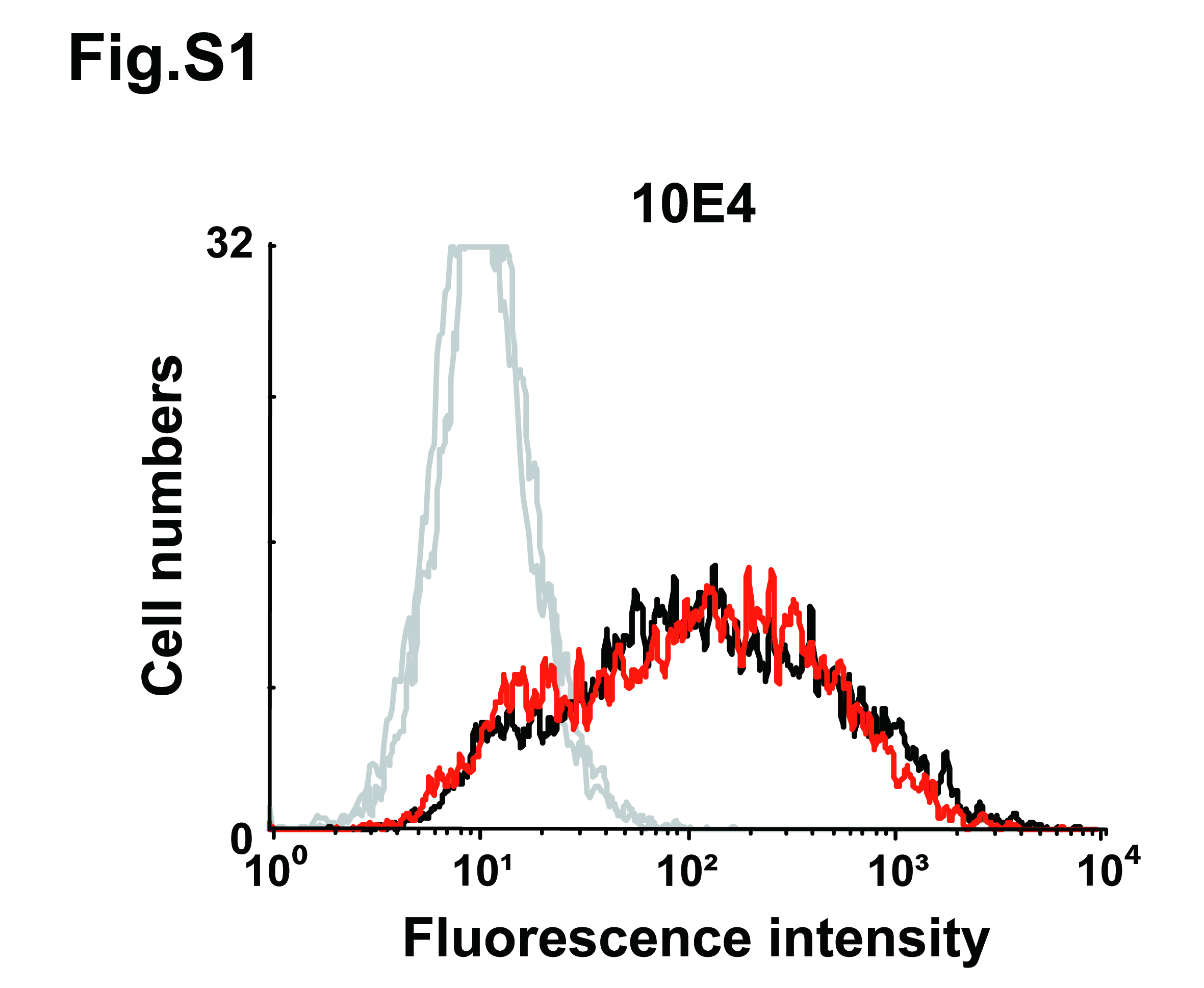

Supplement: Figure S1 — The expression of HS in cells overexpressing 3OST-5 . FACS analysis using the anti-HS antibody 10E4 (black line, control cells; red line, cells overexpressing 3OST-5). The gray line shows the result obtained for cells not treated with primary antibody. (TIF) [file pone.0043440.s001.tif]

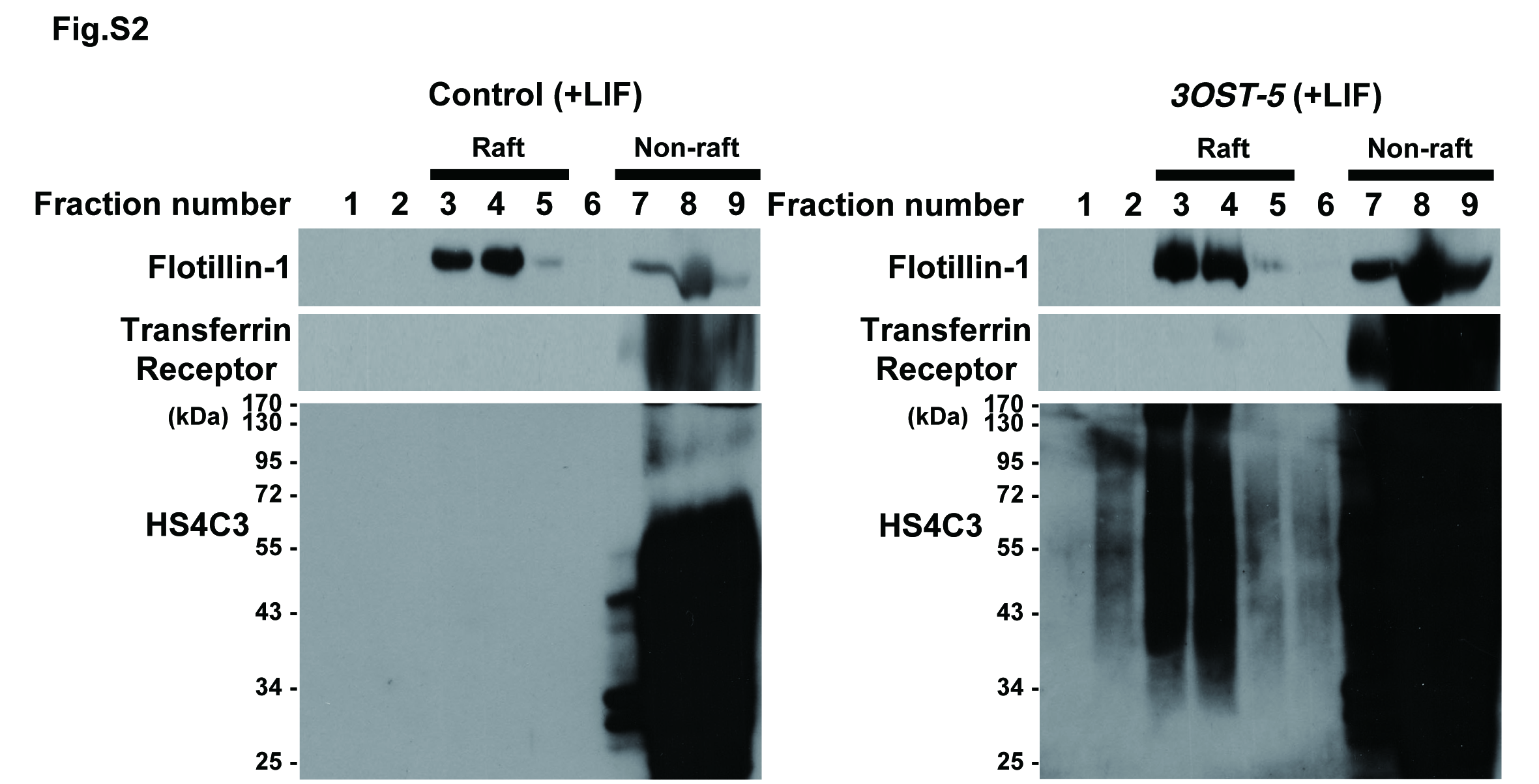

Supplement: Figure S2 — HS4C3-binding epitope localized in lipid rafts in cells overexpressing 3OST-5 . Western blot analysis of raft and non-raft fractions using anti-Flotillin-1 (raft), anti-transferrin receptor (non-raft), and HS4C3 antibodies. Representative results are shown. (TIF) [file pone.0043440.s002.tif]

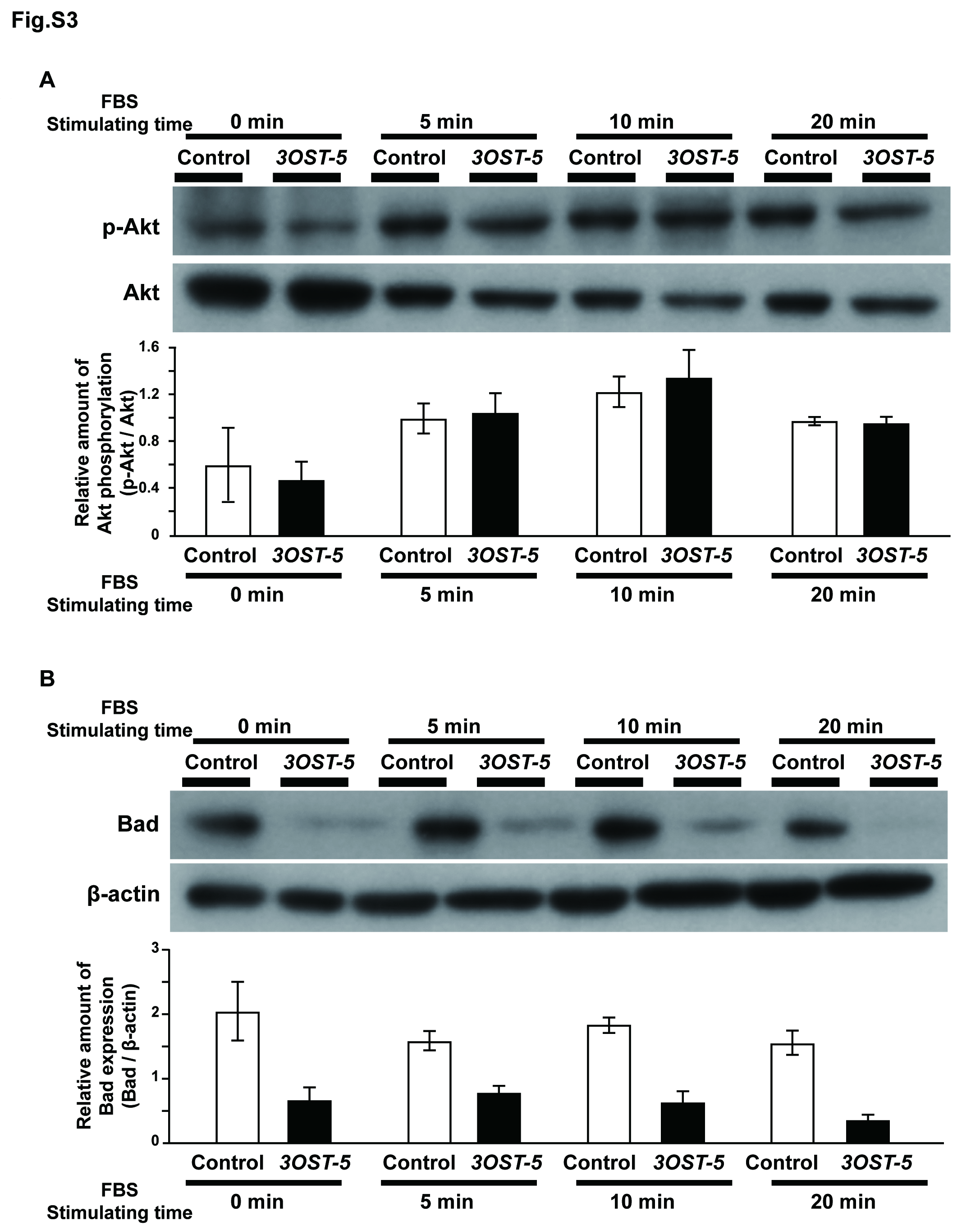

Supplement: Figure S3 — Mitochondrial pathways mediated by survival factors are not affected in cells overexpressing 3OST-5 . (A) and (B) Western blot analysis, using antibodies against p-Akt, Akt, and Bad, of cells stimulated with FBS. The histograms show mean densitometric readings ± SD for the ratio p-Akt/Akt or Bad/β-actin. Three independent experiments were performed, and representative results are shown. (TIF) [file pone.0043440.s003.tif]

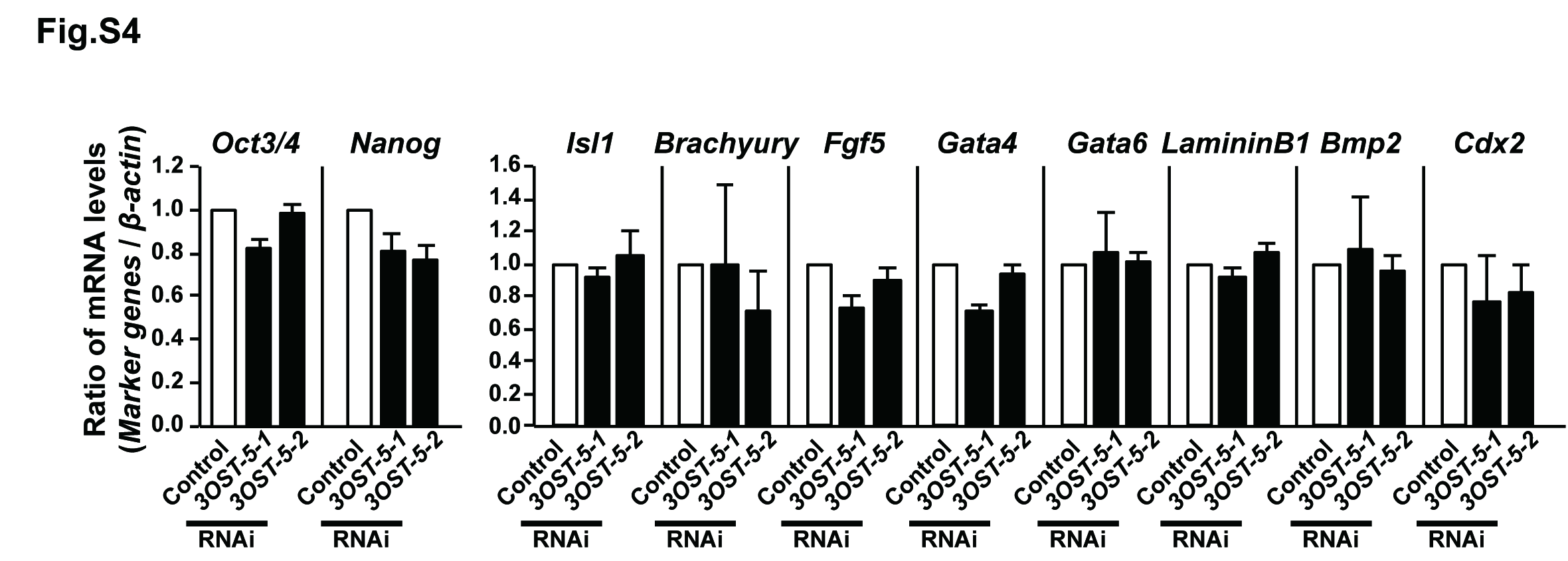

Supplement: Figure S4 — Pluripotency was maintained in stable 3OST-5 knockdown cells. Real time PCR analysis of markers of the undifferentiated and differentiated states in stable 3OST-5 knockdown cells. The values shown are means ± SD after normalization against control cells (arbitrary value = 1). Three independent experiments were performed. *, P<0.01. (TIF) [file pone.0043440.s004.tif]

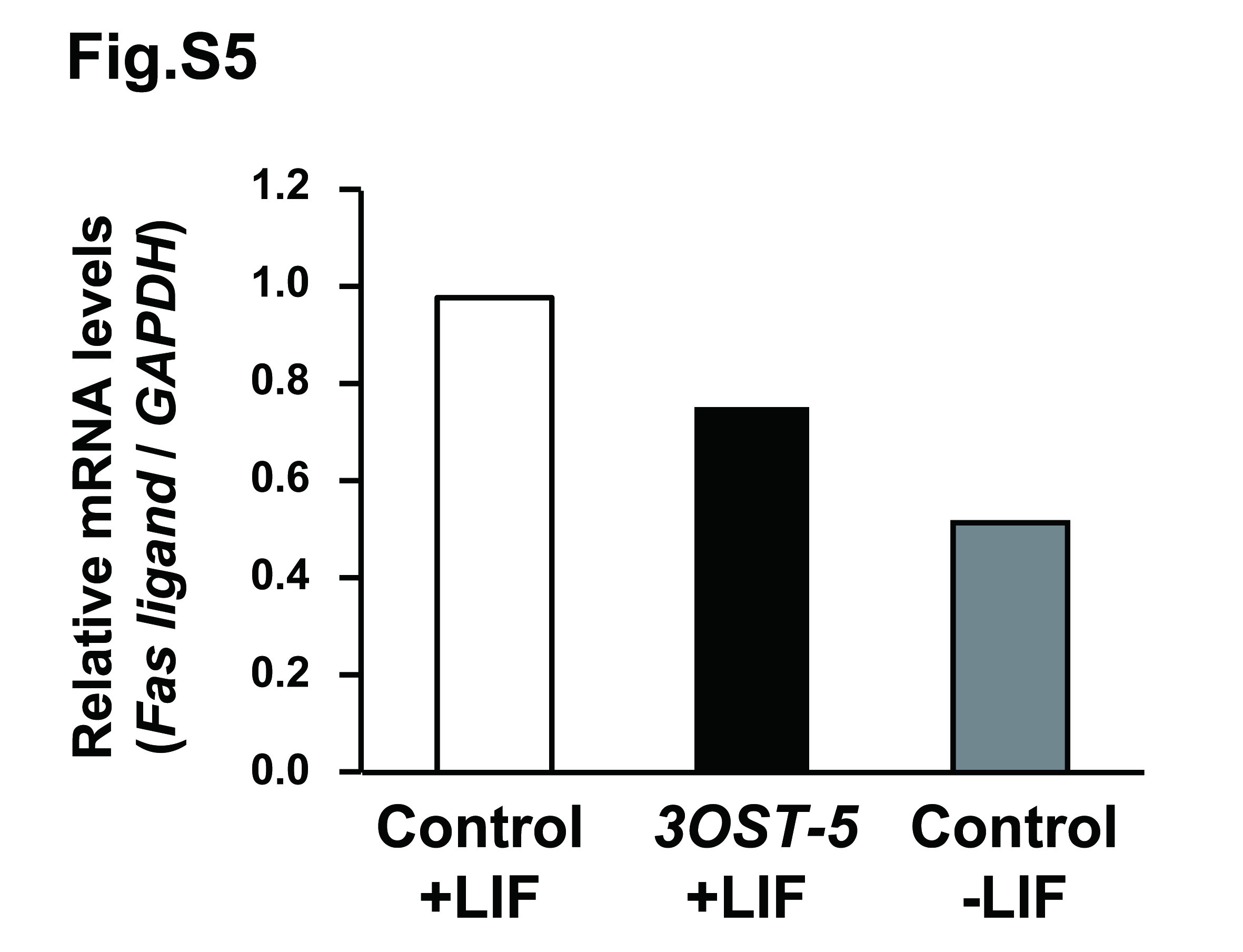

Supplement: Figure S5 — The expression of Fas ligand was not increased in cells overexpressing 3OST-5 . RT-PCR analysis of the expression of Fas ligand in cells overexpressing 3OST-5. GAPDH, glyceraldehyde-3-phosphate dehydrogenase. (TIF) [file pone.0043440.s005.tif]

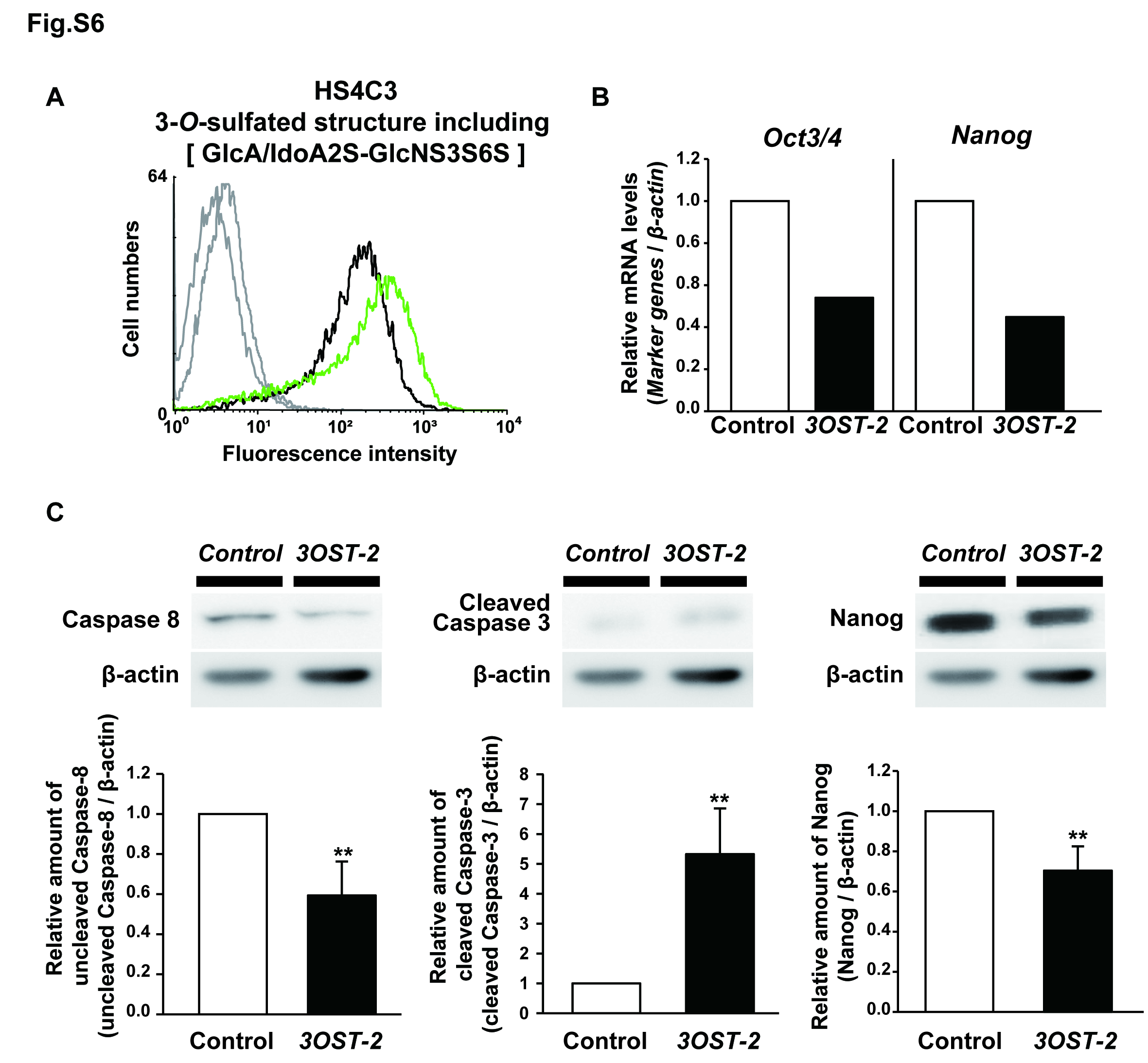

Supplement: Figure S6 — The overexpression of 3OST-2 also activated Fas signaling and induced the differentiation of mESCs. (A) FACS analysis, using the HS4C3 antibody, of mESCs at 2 days after transfection with the 3OST-2 expression construct (black line, control cells; green line, cells overexpressing 3OST-2). The gray line shows the result obtained from cells not treated with primary antibody. (B) Real time PCR analysis of markers of the undifferentiated state in cells overexpressing 3OST-2. The values were normalized against control cells (arbitrary value = 1). (C) western blot analysis using an antibody against uncleaved caspase-8, cleaved caspase-3, or Nanog. The histograms show mean densitometric readings ± SD after normalization against control cells (arbitrary value = 1). **, P<0.05. Three independent experiments were performed. (TIF) [file pone.0043440.s006.tif]

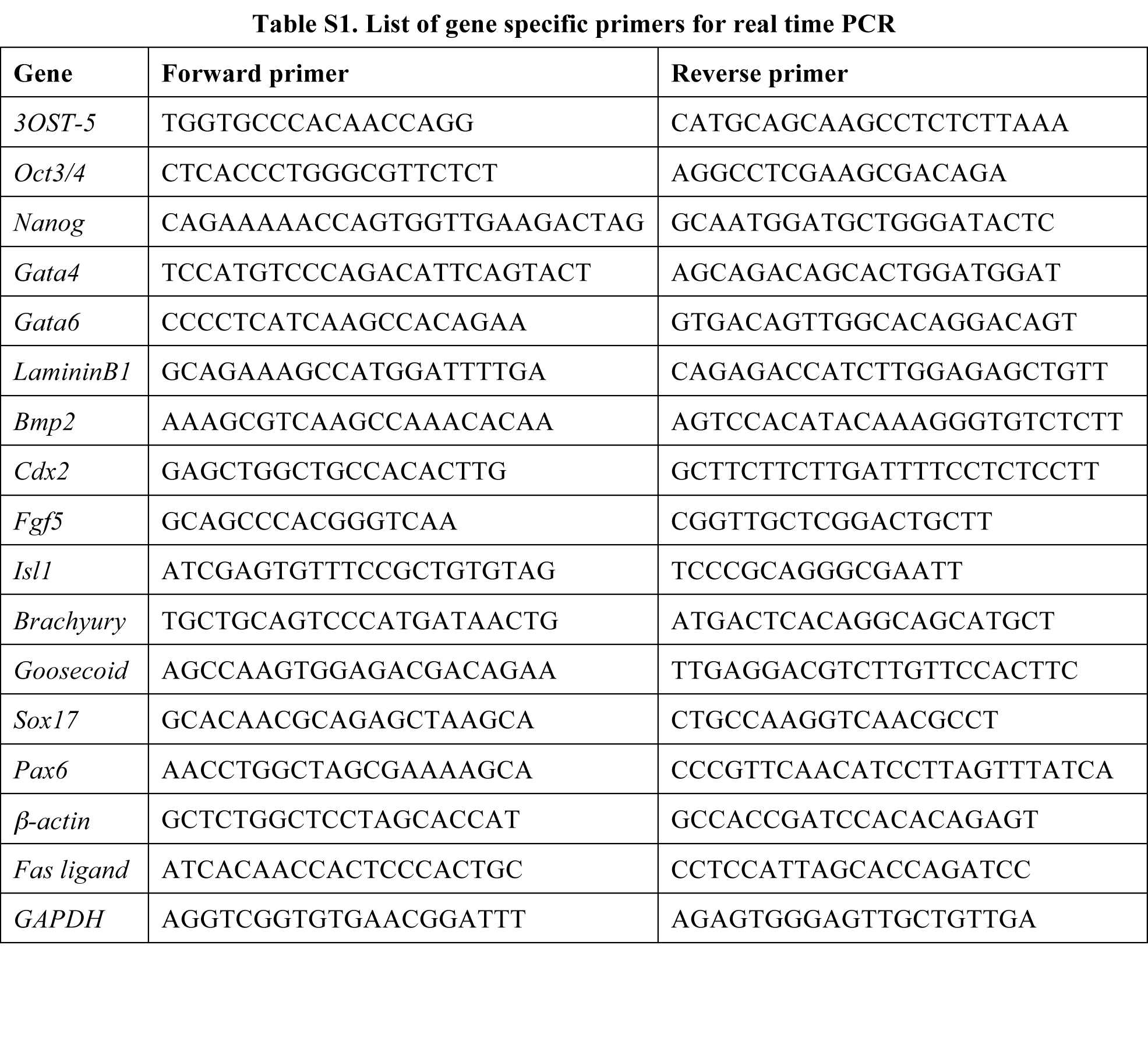

Supplement: Table S1 — List of gene specific primers for real time PCR. (TIF) [file pone.0043440.s007.tif]

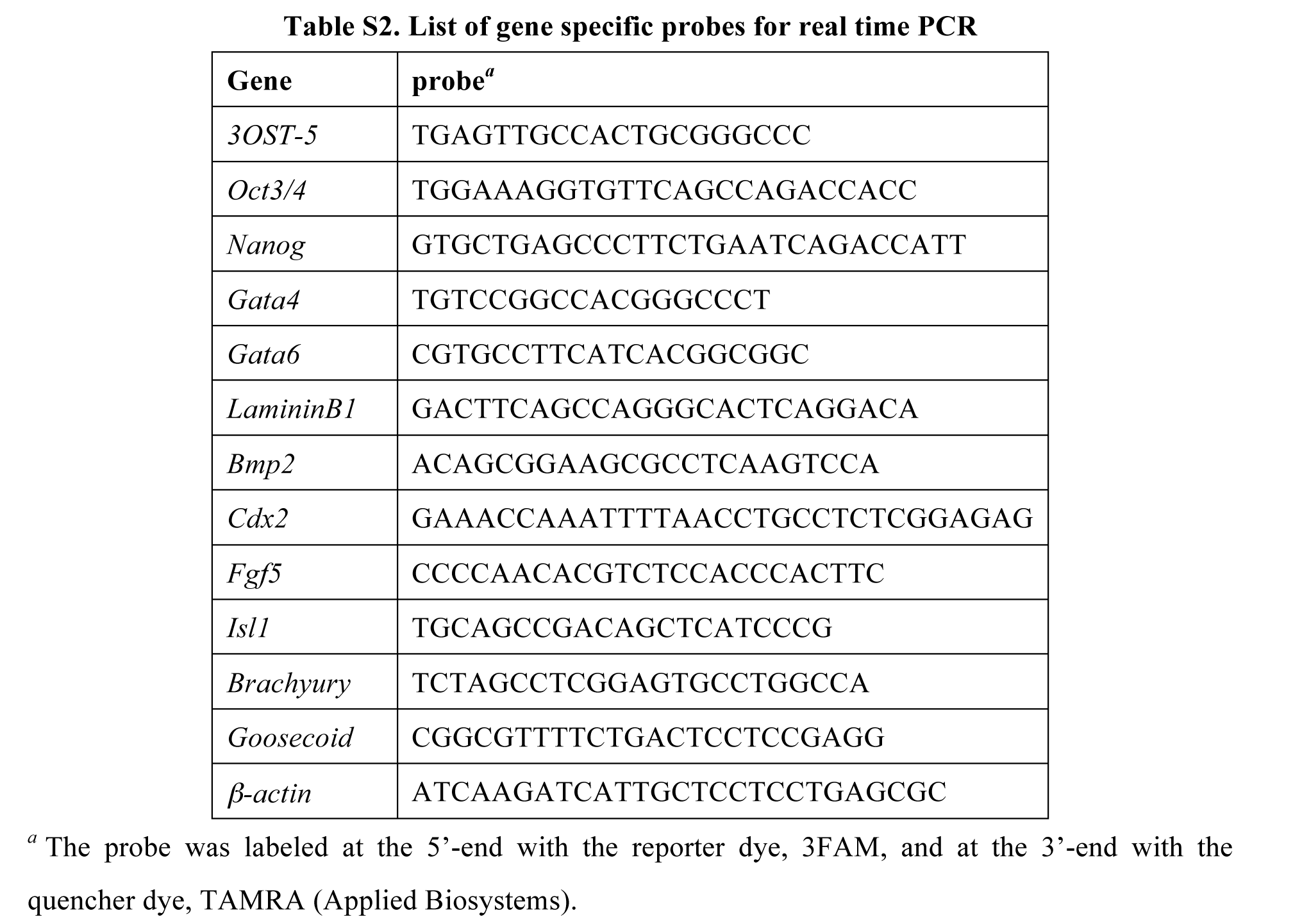

Supplement: Table S2 — List of gene specific probes for real time PCR. (TIF) [file pone.0043440.s008.tif]
